# Emotion-related impulsivity and risky decision-making: A systematic review and meta-regression

**DOI:** 10.1016/j.cpr.2022.102232

**Published:** 2022-11-28

**Authors:** Matthew V. Elliott, Sheri L. Johnson, Jennifer G. Pearlstein, Daniela E. Muñoz Lopez, Hanna Keren

**Affiliations:** a University of California, Berkeley, Berkeley, CA, United States of America; b Azrieli Faculty of Medicine, Bar-Ilan University, Safed, Israel

**Keywords:** Emotion, Impulsivity, Urgency, Risky decision-making, Meta-regression

## Abstract

Emotion-related impulsivity, the trait-like tendency toward regrettable behavior during states of high emotion, is a robust predictor of internalizing and externalizing psychopathology. Despite substantial evidence that emotion-related impulsivity is important transdiagnostically, relatively little is known about its cognitive correlates. This systematic review and meta-regression investigates one such candidate, risky decision-making. We analyzed 195 effect sizes from 51 studies of 14,957 total participants, including 105 newly calculated effect sizes that were not reported in the original publications. The meta-regression demonstrated evidence for a small, positive relationship of emotion-related impulsivity with behavioral indices of risky decision-making (ß = 0.086). Effects generalized across sample age, gender, Positive versus Negative Urgency, and clinical versus nonclinical samples. The average effect size varied by task type, with stronger effects for the Iowa Gambling Task and Delay Discounting Task. Experimental arousal manipulation was nearly a significant moderator, with stress and pharmacological manipulations yielding significant effect sizes. Analyses indicated that publication bias did not skew the current findings. Notwithstanding limitations, the data suggest that risky decision-making is a cognitive domain that relates to emotion-related impulsivity. We conclude with recommendations regarding the specific types of tasks and arousal inductions that will best capture emotion-related impulsivity in future experimental research.

## 1. Introduction

Since the dawn of psychology, scientists and practitioners alike have expressed great concern about impulsivity (Freud, 1947; Guilford and Guilford, 1939). Impulsivity generally refers to maladaptive behaviors that occur without adequate forethought or regard for their consequences; however, a singular definition of impulsivity is difficult to achieve given the vast diversity of behaviors it has been used to describe (Strickland and Johnson, 2021). In modern psychological science, empirical studies of impulsivity have relied primarily on two forms of assessment (Sharma, Markon, and Clark, 2014); those that use self-report instruments to estimate trait-like tendencies, and those that use cognitive tasks to capture experimental measures of poor constraint. Although these measures have all been theorized to tap impulsivity, their coherence is tenuous. Influential self-report measures, such as the Barratt Impulsiveness Scale and UPPS Impulsive Behavior Scale, have shown that impulsivity can be reliably separated into multiple dimensions (Patton, Stanford, and Barratt, 1995; Whiteside and Lynam, 2001). Factor analyses of tasks used to test prominent cognitive theories of impulsivity, such as response inhibition, delay of gratification, and risk-taking, have also not found evidence for a unified impulsivity factor (Reynolds, Ortengren, Richards, and De Wit, 2006; Sharma et al., 2014). Furthermore, the self-report and laboratory measures of impulsivity have correlated weakly in many cases (Cyders and Coskunpinar, 2011; Sharma et al., 2014). These findings have led to a focus on studying separate dimensions of impulsivity.

Perhaps the most influential dimensional model of impulsivity is the UPPS model (Whiteside and Lynam, 2001). The original model was created using factor analysis and was composed of four dimensions: Urgency, (Lack of) Planning, (Lack of) Perseverance, and Sensation Seeking. Confirmatory factor analyses replicated the UPPS model and extended it to include a fifth dimension, Positive Urgency (Cyders et al., 2007; Whiteside, Lynam, Miller, and Reynolds, 2005). The (Negative) Urgency and Positive Urgency scales both capture tendencies toward unconstrained, regrettable speech and behavior during states of high emotion (Carver and Johnson, 2018). (Negative) Urgency items describe contexts involving negative emotions, whereas Positive Urgency items describe contexts involving positive emotions. Although the (Negative) Urgency and Positive Urgency scales are still often measured separately, they correlate so highly that they form a higher order factor across large-scale studies (Carver, Johnson, Joormann, Kim, and Nam, 2011; Cyders and Smith, 2007; Sperry, Lynam, and Kwapil, 2018), suggesting that they fit together in a dimension that is agnostic to valence. Accordingly, we prefer the term emotion-related impulsivity to denote this trait-like tendency to respond impulsively to positive and negative emotions. All of the UPPS self-report scales have strong psychometric properties, including high test-retest reliability and high correspondence with parent-report and interview-based measures (Cyders and Smith, 2007; Zapolski and Smith, 2013; Zapolski, Stairs, Settles, Combs, and Smith, 2010). The strengths of the UPPS model have led to its use in thousands of empirical publications over the past two decades.

Within this vast literature, emotion-related impulsivity has emerged as a critical construct in clinical psychology and public health. Emotion-related impulsivity has been robustly tied to internalizing and externalizing psychopathology, including substance use problems, aggression, suicidality, borderline personality disorder, depression, and disordered eating, across 115 studies (Berg, Latzman, Bliwise, and Lilienfeld, 2015), with an effect size, *r* = .34, considerably larger than the effect sizes for other forms of impulsivity measured by the UPPS, which are consistently at or below *r* = .14. In separate meta-analyses, emotion-related impulsivity has shown moderate effect sizes with bulimia nervosa, *r* = .38, across 50 studies, compared with *r* < .20 for other UPPS scales (Fischer, Smith, and Cyders, 2008), and with non-suicidal self-injury, *d* = .59, again surpassing other UPPS scales, *d* < .32 across 17 studies (Hamza, Willoughby, and Heffer, 2015). The effect sizes observed for emotion-related impulsivity are in the range of those observed for other major psychopathology risk factors, such as neuroticism against dimensional measures of depression, *r* = 0.42 (Malouff, Thorsteinsson, and Schutte, 2005).

Multiple other lines of research have validated the clinical import of emotion-related impulsivity. A growing body of longitudinal work has built upon cross-sectional research and suggests that emotion-related impulsivity can predict a more severe onset and course of substance use problems, eating disorders, risky sexual behaviors, and self-harm (Kaiser, Bonsu, Charnigo, Milich, and Lynam, 2016; Pearson, Combs, Zapolski, and Smith, 2012; Riley, Combs, Jordan, and Smith, 2015; Riley, Rukavina, and Smith, 2016; Zapolski, Cyders, and Smith, 2009). The scale has been shown to predict greater increases in alcohol use after experimental inductions of positive and negative mood states (Cyders, Zapolski et al., 2010; VanderVeen et al., 2016). In a daily diary study, the (Negative) Urgency scale was shown to predict greater increases in self-harm as negative mood states increased (Bresin, Carter, and Gordon, 2013). Parent and interview-based measures of emotion-related impulsivity have been shown to have similarly strong associations with psychopathology (Zapolski and Smith, 2013), which suggests that effects do not simply reflect biases in self-ratings. Last, there is evidence that emotion-related impulsivity is heritable, which increases the likelihood of inter-generational effects (Gustavson, Miyake, Hewitt, and Friedman, 2014; Sanchez-Roige et al., 2019). Together, this body of work provides additional validation of the scale, and indicates that it has strong, transdiagnostic predictive validity.

The widespread reports of elevated emotion-related impulsivity in clinical populations have prompted experimental studies to determine the basic cognitive processes that correlate with this trait (e.g., Cyders et al., 2010; Pearlstein, Johnson, Modavi, Peckham, and Carver, 2019). Understanding the cognitive correlates of emotion-related impulsivity would have at least three main benefits. One, it would allow for more precise, controlled, and cost-efficient research using task-based, functional neuroimaging, which in turn would advance our understanding of the brain systems involved. Two, it would provide a foundation to experimentally study the contexts in which emotion-related impulsivity arises. While the regrettable speech and actions that characterize emotion-related impulsivity may be difficult to induce in the laboratory, isolated cognitive correlates could be studied in experiments that manipulate socioemotional contexts – like arousal, peer rejection, or hunger – and identify areas of special vulnerability. Ultimately, better knowledge of cognitive correlates, neural correlates, and contextual triggers could set the stage for the development and improvement of psychological interventions for emotion-related impulsivity.

One might expect that emotion-related impulsivity would relate to greater emotional reactivity (Clark, 2005) and indeed, the Negative and Positive Urgency scales have been found to correlate with trait measures of neuroticism (Cyders and Smith, 2008; Sharma et al., 2014; Whiteside and Lynam, 2001). Many findings, however, do not fit with the idea that greater emotional reactivity is driving the effects of emotion-related impulsivity. For example, those with higher emotion-related impulsivity do not show greater psychophysiological or behavioral emotional reactivity in laboratory studies with experimental manipulations of mood (Johnson et al., 2017; Owens, Amlung, Stojek, and MacKillop, 2018; Wise, Phung, Labuschagne, and Stout, 2015). Emotion-related impulsivity also correlates with psychopathology when controlling measures of emotional reactivity and neuroticism (Cyders et al., 2010; Cyders and Coskunpinar, 2010; Cyders and Smith, 2008).

Response inhibition, the ability to override prepotent actions, is one cognitive correlate of emotion-related impulsivity. Although effect sizes of response inhibition and emotion-related impulsivity were larger in clinical samples (Bagge, Littlefield, Rosellini, and Coffey, 2013; Dekker and Johnson, 2018; Rochat, Beni, Annoni, Vuadens, and Van der Linden, 2013), effects have been small in nonclinical samples (Cyders and Coskunpinar, 2012; Gay, Rochat, Billieux, d’Acremont, and Van der Linden, 2008), and most variance in emotion-related impulsivity remains unexplained by response inhibition (Johnson, Tharp, Peckham, Sanchez, and Carver, 2016). Since fast, reflexive processes like emotional reactivity and response inhibition do not account for most of the variance in emotion-related impulsivity, more deliberative cognitive processes may also be involved.

One such candidate is risky decision-making, which involves modulation of goal pursuit based on situational risks or costs. Three functional MRI studies have replicated a correlation of emotion-related impulsivity with increased anterior insula activation during decision-making tasks involving risk (Smith et al., 2018; Xiao et al., 2013; Xue, Lu, Levin, and Bechara, 2010). The consistency of these neuroimaging findings supports the idea that risky decision-making is related to emotion-related impulsivity; however, syntheses of the studies using behavioral measures have been less clear. In one meta-analysis, Negative Urgency was not correlated with measures of delay discounting, which tests decision-making for smaller, more proximal rewards versus larger rewards at the cost of larger temporal delays (r = 0.01, Cyders and Coskunpinar, 2011). The meta-analysis included multiple studies employing delay discounting questionnaires but only two studies had included laboratory delay discounting measures with Negative Urgency (Manwaring, 2009; Verdejo-García, Lozano, Moya, Alcázar, and Pérez-García, 2010), and only one effect size with Positive Urgency was available (*r* = .13, *p* = .11, Verdejo-García et al., 2010). Considerable cross-study heterogeneity in effect sizes was observed, but the dataset was too small to examine moderation by task format or type. A second meta-analysis resulted in a small correlation of the Urgency scale with the Iowa Gambling Task (*r* = .09), and a stronger correlation with delay discounting tasks that offered actual monetary incentives (*r* = .24), but not when such incentives were not present (*r* = .03) (Sharma et al., 2014). Given the limited size of the literature at the time, these findings were drawn from small numbers of effects (k = 2 in some cases). These two key meta-analyses did not lead to strong conclusions regarding risky decision-making as a correlate of emotion-related impulsivity, given the heterogeneity they uncovered.

Fortunately, dozens of studies with measures of risky decision-making and emotion-related impulsivity have been published since the most recent meta-analysis, opening the door for us to test multiple key moderators. Task type is one such potential moderator, as risky decision-making tasks are evidently not monolithic. Research has shown that a common measure of risky decision-making, the Balloon Analogue Risk Task, did not correlate with other measures of decision-making and risk-taking, such as the Iowa Gambling Task or delay of gratification indices (Sharma et al., 2014). Conceptual divisions also exist. For example, gambling tasks measure decision-making when consequences are uncertain, whereas the consequences of choices in delay discounting tasks are certain. Therefore, the types of risk or cost associated with decisions in these tasks may be important to tease apart.

Beyond examining task domains, mood state may moderate the relationship between emotion-related impulsivity and risky decision-making. Those with tendencies toward emotion-related impulsivity show more symptomatic behavior after positive and negative mood inductions (Cyders et al., 2010; Manasse et al., 2018; VanderVeen et al., 2016), and two studies found that response inhibition is impaired after mood inductions for those with emotion-related impulsivity (Dekker and Johnson, 2018; Johnson et al., 2016). Research on mood state as an influence on emotion-related impulsivity and risky decision-making has yielded mixed results. In one study, researchers measured risk-taking on the Balloon Analogue Risk Task before and after a positive mood induction. Positive Urgency predicted a greater increase in risky behavior after the positive mood induction as compared to pre-mood induction (Cyders et al., 2010), but these findings were not replicated in a second study (Johnson et al., 2016). We aim to synthesize the extant research considering the role of mood in shaping whether those with high emotion-related impulsivity demonstrate risky decision-making.

Given that emotion-related impulsivity relates to positive and negative emotions (which share the experience of arousal), some work has investigated the function of physiological arousal. In one study examining trial-level performance within a response inhibition task, emotion-related impulsivity moderated the effect of arousal (as measured using pupil dilation) on accuracy. That is, among participants with lower emotion-related impulsivity, accuracy was positively correlated/ increased with higher arousal; whereas among participants with higher emotion-related impulsivity, accuracy was negatively correlated/ decreased with higher arousal (Pearlstein et al., 2019). Furthermore, higher Positive Urgency related to stronger delay discounting (i. e., preference for smaller, more immediate rewards) for imaginary sexual activity (Carrier Emond, Gagnon, Nolet, Cyr, and Rouleau, 2018). This fits with the idea that emotion-related impulsivity is particularly likely to lead to unconstrained behavior and decision-making during periods of higher arousal. Accordingly, we systematically review and synthesize studies that consider arousal manipulations as potential moderators of the link between emotion-related impulsivity and risky decision-making.

As the literature on emotion-related impulsivity has progressed, several other trends have emerged that warrant systematic review in the current study. One, correlations of emotion-related impulsivity with response inhibition performance appear more robust in clinical compared to nonclinical student or community samples (Johnson et al., 2016). Two, men tend to engage in more risky decision-making than do women (Byrnes, Miller, and Schafer, 1999; Charness and Gneezy, 2012), and three, adolescents and young adults engage in more risky decision-making than do older adults (Steinberg, 2004; Willoughby, Heffer, Good, and Magnacca, 2021). Four, the Positive Urgency and Negative Urgency scales are highly correlated (Cyders and Smith, 2007) and have parallel relationships with many clinical and neuroimaging variables (Johnson, Elliott, and Carver, 2020). Thus, we include sample type (clinical vs. non-clinical), gender, age, and self-report measure (Positive vs. Negative Urgency) as additional moderators. Taken together, we predict that the correlations of emotion-related impulsivity with risky-decision making will be larger in samples that are clinical, have higher proportions of young people, and higher proportions of men. We expect no significant difference between effect sizes for Positive Urgency and Negative Urgency.

Previous meta-analyses broadly investigating the covariance of impulsivity measures offered the idea that types of trait impulsivity may differ in their cognitive correlates (Cyders and Coskunpinar, 2011; Sharma et al., 2014). There now exists the opportunity to extend this work with an eye toward high-priority constructs in clinical psychology. Indeed, the goal of this review and meta-regression is to rigorously investigate the relation between emotion-related impulsivity – a dimension of trait impulsivity of great consequence for clinical psychology – and risky decision-making – an oft studied, but not yet synthesized, cognitive correlate – with special focus on key experimental design and sample moderators.

## 2. Method

The study protocol and hypotheses for this systematic review and meta-regression were pre-registered on the Open Science Framework (OSF) website on May 29, 2020. Along with the registration (https://osf.io/r8zu7/?view_only=3d9a409bc22d47a196347d12c68f862e), all data and analysis scripts have been posted on OSF (https://osf.io/n8a3v/?view_only=6db27a14d316420fbd1cbb9648757f0d). We note where we changed methods after pre-registration. For example, although we hoped to examine curvilinear patterns between emotion-related impulsivity and risky decision making, such data was unavailable and could not be incorporated here. We followed the Journal Article Reporting Standards for Quantitative Meta-Analyses (Appelbaum et al., 2018).

### 2.1. Search method

To identify articles that included an emotion-related impulsivity measure and a decision-making task, we conducted a literature search on July 5, 2022. We used five search engines: PsychInfo, PubMed, Scopus, Web of Science, and psyarXIV. For PsychInfo, our search terms were: (upps OR UPPS-P OR TM(urgency) OR “emotion-related impulsiv*” OR “three factor impulsiv*” OR “emotion-triggered impulsiv* OR “emotion-induced impuls*” OR “emotion* impulsiv*”) AND (risky OR “probability discounting” OR “intertemporal choice” OR “BART” OR “balloon analogue” OR “gambling task” OR “iowa gambling” OR “IGT” OR “delay discount* OR “temporal discount*” OR “choice behavior” OR “information sampling” OR “reflection impulsivity” OR “delay of gratification” OR “risk-taking” OR “risk taking”). Corollary searches were conducted in the other four engines. The searches were limited to articles that were published on or after the year 2001, which is when the UPPS scale and the construct of emotion-related impulsivity were published (Whiteside and Lynam, 2001).

Literature searches identified 863 articles. We searched the cited references of these articles and identified 25 additional studies, which were not identified by the literature searches but were potentially relevant to our review. We also wrote the corresponding authors of the articles that were included (see below) to ask for any additional publications and preprints, which yielded six additional articles and preprints. After removing duplicates, the pool contained 621 articles and preprints.

### 2.2. Inclusion and exclusion

**Fig. 1.**
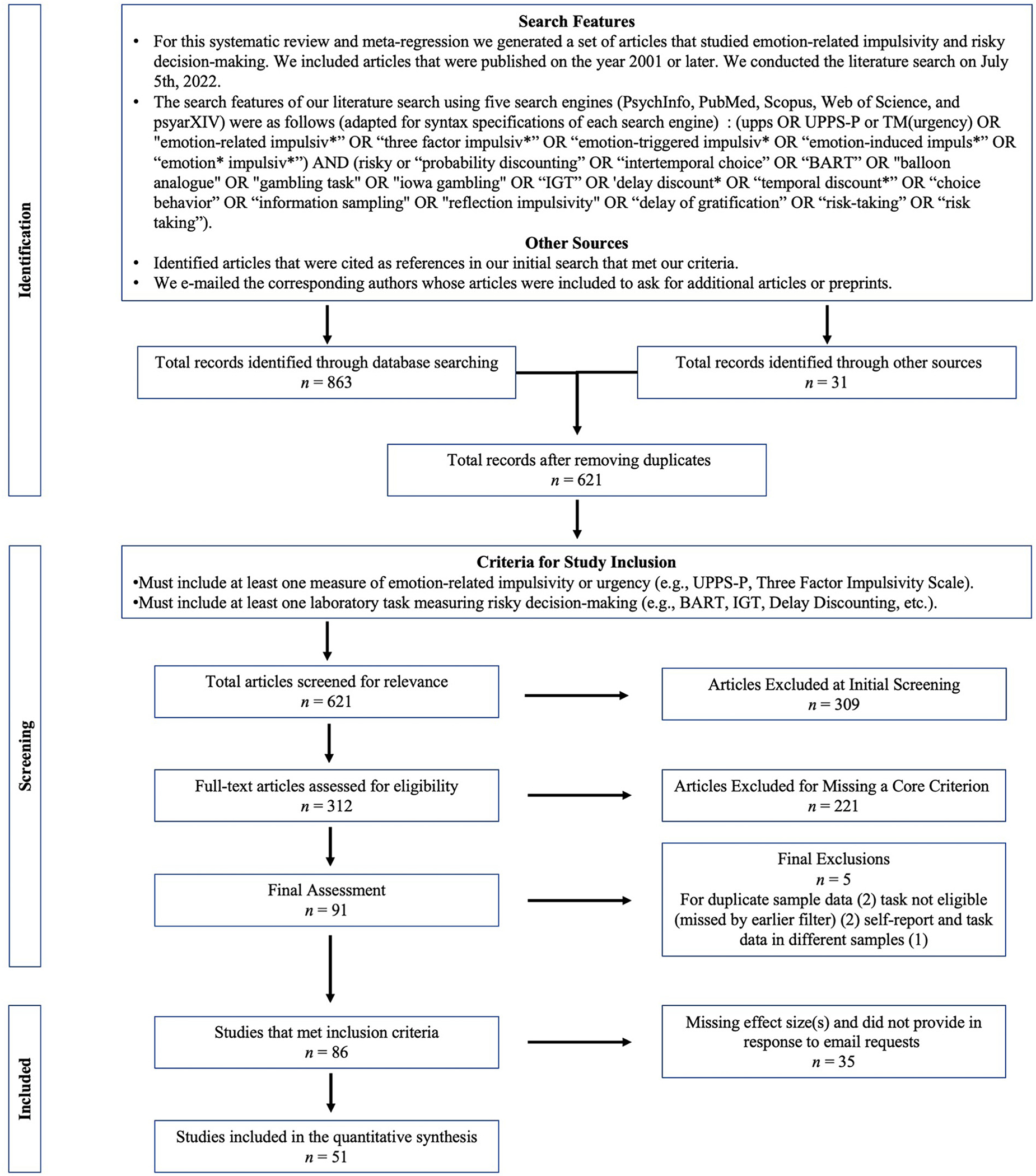
PRISMA-style flow diagram showing the search criteria, selection of studies, inclusion criteria, and exclusion rationale of studies for meta-regression.

Fig. 1 illustrates the steps by which articles were excluded. To be included, journal articles, manuscripts, and pre-prints were required to report empirical data and include both a measure of emotion-related impulsivity and a behavioral measure of decision-making. Although we considered including self-rated measures of risky decision-making, we see one advantage of behavioral measures being the relevance for imaging and experimental research. Accordingly, we focus on behavioral tasks here. The fourth author, D.M.L, conducted an initial screening for the inclusion of an emotion-related impulsivity measure, which led to the exclusion of 309 articles. M.E., J.P., and S.J. conducted independent blind reviews of the remaining 312 articles to determine inclusion in the present meta-analysis. Interrater reliability for study inclusion was *κ* = 0.93. All discrepancies were discussed among the three authors who conducted blind reviews, and inclusion was determined by consensus. 221 articles were excluded for missing a core inclusion criterion, leaving 91 articles. A final pass through the articles led to two exclusions for duplicate sample data, two exclusions for not including a risky decision-making task (should have been excluded earlier), and one exclusion for having not gathered the self-report and task data within the same sample. Of the 86 studies that met full criteria for inclusion, 30 studies reported a total of 90 relevant effect sizes with the corresponding sample sizes and moderator variables in the main text, the supplement, or in an online database. 56 studies did not report any type of statistical test of the relationship between emotion-related impulsivity and decision-making task performance. For these studies, we emailed corresponding authors to request the data needed for inclusion. The authors of 21 studies replied with the requested effect sizes or raw datasets. From these 21 studies we added 105 effects, which are reported here for the first time. The authors of 35 studies did not reply to two attempts at correspondence or responded that they did not have information to share, therefore those studies could not be included. The final set of *n* = 51 studies yielded *k* = 195 effect sizes.

### 2.3. Data extraction

#### 2.3.1. Effect sizes

M.E., S.J. and J.P. extracted all relevant effect sizes for the strength of the relationship between emotion-related impulsivity and risky decision-making. In one exception to including multiple effect sizes from a given study, we chose to use only the first chronological effect reported in a three-wave longitudinal study (Booth et al., 2017). Most effect sizes were bivariate correlation coefficients, however, partial correlation coefficients from one study were also included (Xiao et al., 2009). Where raw datasets were provided, we calculated Pearson’s *r* for each relevant effect. We converted correlation coefficients to Fisher’s *z* to control for undesirable statistical properties in correlation coefficient distributions.

#### 2.3.2. Variable operationalization and coding

We coded study features in the following steps. First, M.E., J.P., and S.J. each coded a third of the included studies independently. Second, M. E. and J.P. reviewed the coded studies, made coding adjustments as needed, and recorded the rationale for the adjustments. Third, S.J. and M.E. checked all studies, including reasons for any coding adjustments. Interrater reliability of coding across all variables was very high, *κ* = 0.99. Rare disagreements, such as the coding of a sample as clinical vs. non-clinical, were resolved through discussion and consensus.

To assess descriptive features, we extracted the sample size and coded publication status. As proxies for sample diversity, we coded the country where the study took place and racial identity. The race variable was operationalized as the percentage of study participants that identified as white; we chose this index due to heterogeneity in how race was reported.

Next, we coded the pre-registered potential moderators. For the gender variable, the proportion of men and women was operationalized as the percentage of women in the sample. The age variable was operationalized as the mean age of participants in the sample. For the sample type variable, samples were coded as (1) clinical or (0) non-clinical (which included community and undergraduate samples). We also coded the self-report measure used in each effect size – Positive Urgency (PU) or Negative Urgency (NU). To operationalize task-type in the meta-regression, we assigned each risky decision-making task an abbreviated code (e.g., IGT for Iowa Gambling Task; Table 1). In post-hoc analyses, we grouped effects from all “gambling tasks,” which were defined as tasks where participants wagered something of value on a trial with an uncertain outcome. The following tasks were re-coded as “Gambling” in this secondary analysis: Cambridge Gambling Task (CGT), Columbia Card Task (CCT), Cups Task, Game of Dice Task (GDT), Holt-Laury Risk Task (Holt), Iowa Gambling Task (IGT), Risky Gains Task (RGT), and Verbruggen Gambling Task (VGT).

The inclusion and type of arousal or mood induction were coded using the following categories: (1) no manipulation of arousal or mood; (2) imaginary sexual incentive; (3) peer supervision; (4) pharmacological manipulation of arousal (e.g., yohimbine); (5) stress induction; (6) positive mood/affect induction. Separately, we coded whether monetary incentives were provided based on performance. Tasks in which participants only earned points or fictitious money were coded as not providing monetary incentives.

Some studies included multiple tasks and/or multiple indices of risky decision-making per task. For these studies, we analyzed data for each respective index. One exception was the Balloon Analogue Risk Task. Because balloons are set to explode at random, the number of exploded balloons is less directly reflective of participant behavior than the number of pumps on unexploded balloons (Lejuez, Aklin, Zvolensky, and Pedulla, 2003). Therefore, we prioritized the number of pumps on unexploded balloons and included the number of exploded balloons only when number of pumps was not available (*k* = 2). For each index of risky decision-making where high scores represented low risk-taking, we reversed effect sizes so that high scores would consistently indicate high risk-taking (e.g., −0.2 changed to 0.2).

We considered multiple standard guidelines for assessing study quality (Valentine, 2019; Viswanathan and Berkman, 2012). Because these are often developed for medical and intervention science, frequently coded indices, such as inter-rater reliability, blinding, random assignment, and inclusion of comparable control groups or placebo were not relevant to the current analyses. In many ways, our studies were already constrained by our requirement to focus on behavioral indices of risky decision-making. Nonetheless, we identified and coded one key indicator of data quality: the number of trials in each risky decision-making task, which can influence task reliability. Beyond data quality, studies differed in two facets of their reporting quality: whether they reported the racial identities of their participants, which may indicate attention to sample representativeness, and in those studies that included an arousal or mood induction, whether they reported data about the success of the manipulation.

### 2.4. Planned analyses

Alpha was set at 0.05 and analyses were conducted using R.

#### 2.4.1. Meta-regression – pooled effect and moderator

The pooled effect size between emotion-related impulsivity and laboratory indices of risky decision-making was estimated using meta-regression. An advantage of meta-regression is the ability to examine moderating effects concurrently in a single model (Pustejovsky and Tipton, 2022). Much like multiple regression in single sample studies, this improves precision in estimating the effect of each moderator on the outcome variable.

Traditional random effects meta-regression assumes independence between effect sizes. Therefore, if the within-study covariance structure is unknown, as is often the case in meta-regression, multiple, non-independent effect sizes from a single study must be pooled. This causes information loss and reduces the ability of the researcher to examine sources of heterogeneity. Robust variance estimation (RVE) is a meta-analytic technique that is robust to dependent effects in meta-analysis and provides valid estimates and standard errors even when the underlying dependence structure is unknown (Tipton, 2013; Tipton, 2015). Because we did not have access to the within-study covariance structures from studies that reported multiple effects, we shifted from our pre-registered plan of using structural equation meta-analysis to RVE meta-regression.

For our core analyses, we used the *robumeta* package to build two correlated effects RVE meta-regression models with assumed within-sample correlations (rho) set to 0.6 (Fisher, Tipton, and Zhipeng, 2017). To test robustness of the findings, we also built equivalent models with rho equal to 0.4 and 0.8.

The first meta-regression was an intercept-only model to estimate the pooled effect size. Studies varied in the number of relevant effect sizes that they reported, and five studies reported more than ten effects and accounted for 80 out of 195 total effect sizes (41%). To determine whether the findings of the original model were skewed by these five studies being over-represented, we tested the intercept-only meta-regression with these studies removed (not pre-registered).

The second meta-regression model included moderator variables of interest – age, gender, task type, arousal manipulation, monetary incentive, sample type, and impulsivity measure type. We followed two guidelines to avoid interpreting underpowered moderators. First, any moderator variables that were missing for the majority of effect sizes were dropped from the meta-regression. Second, per recommendations by Tanner-Smith, Tipton, and Polanin (2016), we did not interpret moderators with fewer than four small-sample corrected degrees of freedom. For categorical moderator variables, any levels that did not have four or more small-sample corrected degrees of freedom were collapsed to conserve data.

Recent advances in the flexibility of RVE meta-regression have allowed for models that simultaneously account for correlated and hierarchical dependence structures in a dataset (Pustejovsky and Tipton, 2022). We built a correlated and hierarchical effects (CHE) model with RVE standard errors using the *metafor* (Viechtbauer, 2010) and *clubSandwich* (Pustejovsky, 2020) packages and the methods described by Pustejovsky and Tipton (2022) to test the robustness of our moderation findings in the case that unforeseen hierarchical dependence structures existed in this dataset.

For each of the models described, the relevant effect sizes (e.g., the z-transformed correlation coefficients) were included and weighted proportionally to their sample variance, which was estimated as an adjusted inverse of the sample size: $V=\frac{1}{n-3}$ (Tanner-Smith et al., 2016). Standard errors were then estimated as the square roots of the variances: $\sqrt{\frac{1}{n-3}}$ (Borenstein, Hedges, Higgins, and Rothstein, 2009).

We estimated heterogeneity of effects using two common metrics - τ^2^ and *I*^*2*^. τ^2^ is an estimate of the between-study variance relative to the within-study variances. *I*^*2*^ describes the percentage of between-study variance that is due to heterogeneity compared to sampling error (Higgins and Thompson, 2002). Substantial heterogeneity (*I*^*2*^ > 50%) may be indicative of meaningful subgroups in the set of effect sizes (Higgins et al., 2019).

#### 2.4.2. Non-hypothesized sample characteristics

In addition to age and gender, which were pre-registered moderated variables included in the meta-regression, we tested the generalizability of effects in relation to three sample characteristics that were not pre-registered – percentage of the sample identifying as white (i.e., Percent White), year of publication, and country of research. These were not hypothesized to moderate the aggregate effect size, and we tested each separately using the correlated effects RVE meta-regression technique described above.

#### 2.4.3. Study quality

We also tested the stability of effects in relation to three data and reporting quality indicators: 1) number of trials in the task, 2) whether the racial identities of the participants in the sample were reported, and 3) whether studies that used arousal manipulations checked their effectiveness. We tested each of these separately using the correlated effects RVE meta-regression technique described above (not pre-registered).

#### 2.4.4. Assessment of publication bias

We investigated potential publication bias in four ways. First, we used a funnel plot to visually inspect the data and examine for skewness in the bivariate distribution of effect size and sample size. Funnel plots reflecting publication bias are asymmetrical and have disproportionately few nonsignificant effects with small sample sizes (Sterne and Harbord, 2004). Second, we conducted a trim and fill analysis (Duval and Tweedie, 2000), using the *meta* package (Balduzzi, Rücker, and Schwarzer, 2019). We clustered the effects by study and chose a random effects model to estimate the number of unpublished null effect sizes, given the distribution of effects included in this meta-regression. Third, in addition to the pre-registered checks, we conducted a P-Curve analysis (http://p-curve.com) to examine the distribution of significant p-values and test for evidence of a true aggregate effect (Simonsohn, Nelson, and Simmons, 2014; Simonsohn, Simmons, and Nelson, 2015). In the case of a true effect, the p-curve is right-skewed with an increased incidence of low significant p-values (*p* < .01) compared to high significant p-values (.04 < *p* < 0.05). Fourth, we conducted a sensitivity analysis using the *PublicationBias* package to determine the severity of publication bias that would have to be present to fully attenuate the aggregate effect (Mathur and VanderWeele, 2020). Severity of publication bias (*η*) was defined as the number of times more likely an affirmative study was to be published than a non-affirmative study and tested at a recommended range of 1 to 200.

## 3. Results

### 3.1. Study characteristics

**Fig. 2.**
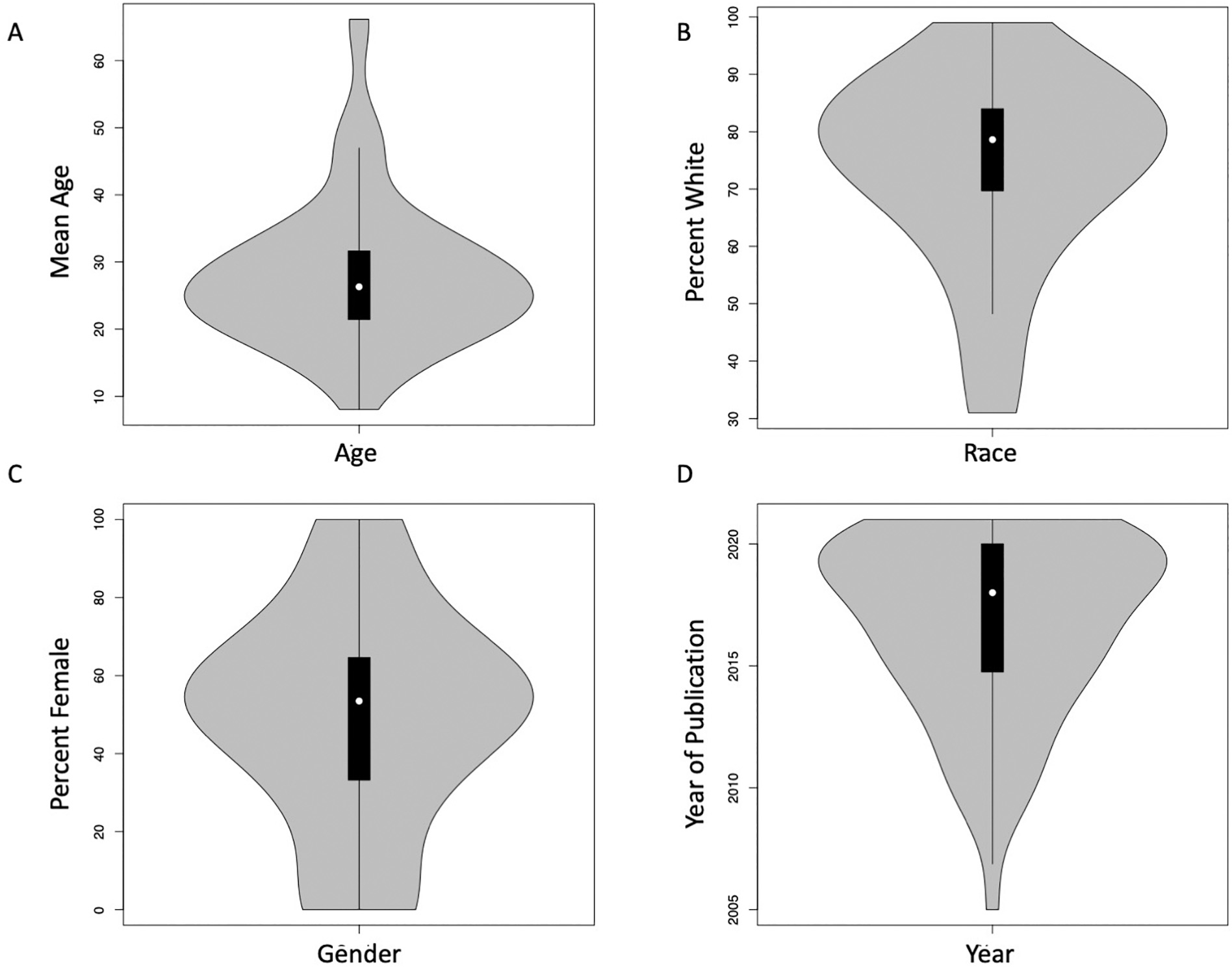
Violin plots of (A) the mean age (B) the percentage identifying as white and (C) the percentage identifying as female (D) the year of publication across all samples included in the meta-regression.

Appendix A includes sample characteristics, moderator variables, and study quality indicators for the *n* = 51 included studies, yielding *k* = 195 effect sizes that reported data from 14,957 participants (age *M* = 32.94, *SD* = 10.62). 90 effect sizes were extracted from an article or supplement, and we generated 105 novel effect sizes from studies that had the necessary data but had not reported on emotion-related impulsivity and risky decision-making. Of the 195 effect sizes included, only five were covered in previous meta-analyses (Cyders and Coskunpinar, 2011; Sharma et al., 2014). The number of included effect sizes per study ranged from a minimum of one to a maximum of twenty (median = 2). Five studies reported ten or more effect sizes; however, most effect sizes came from studies that reported four or fewer (Appendix B.1). Figure 2 illustrates the distributions of the continuous sample characteristic variables – Age, Percent Female, Percent White, and Year of Publication. Table 2 captures the numbers of studies (*n*) and effect sizes (*k*) of the categorical moderator variables – Sample Type, Arousal Manipulation, Impulsivity Measure, and Task Type. Four tasks had sufficient data to be included as independent categories of the Task Type variable – Delay Discounting Task (*n* = 23, *k* = 83), Balloon Analogue Risk Task (*n* = 13, *k* = 27), Iowa Gambling Task (*n* = 9, *k* = 24), and Information Sampling Task (*n* = 4, *k* = 14). To conserve data for the tasks that were used in fewer than four studies, the remaining twelve tasks were included with the task type variable re-coded as “other” (*n* = 13, *k* = 47). Because several variants of gambling tasks were included in the “other” category, we conducted post-hoc analyses of a consolidated category of gambling tasks. No individual type of arousal manipulation achieved four small-sample corrected degrees of freedom, so we reduced our six categories to examine whether there was an arousal manipulation (1) or not (0).

### 3.2. Intercept-only meta-regression

**Fig. 3.**
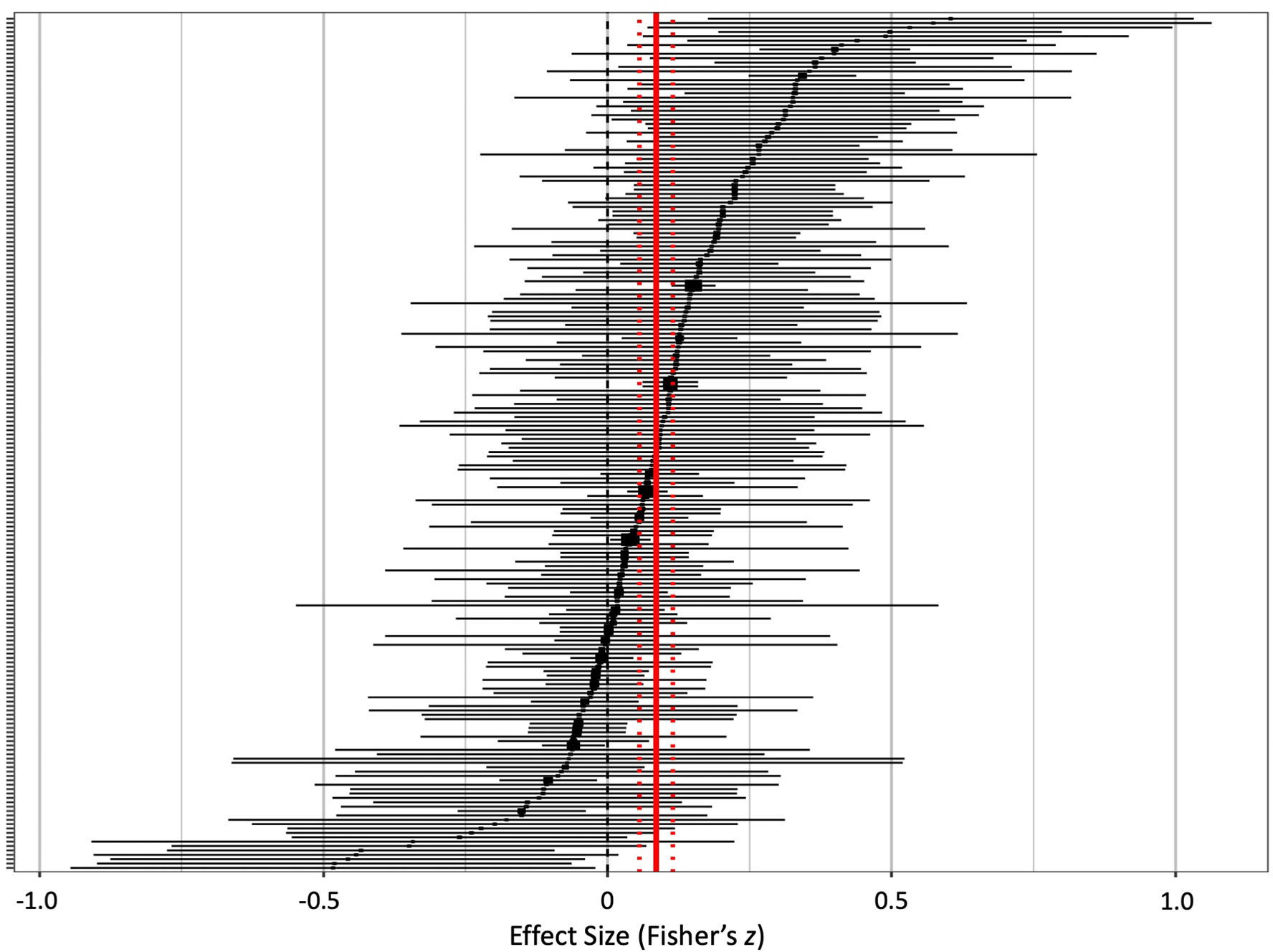
Forest plot for all included effects of risky decision-making and emotion-related impulsivity. Point estimates (squares) with 95% confidence intervals (horizontal lines) are sorted from strongest positive (top) to strongest negative (bottom). Positive effect sizes are those that found high risky decision-making correlating with high emotion-related impulsivity. Solid vertical line represents the pooled effect, estimated by intercept-only meta-regression. Dashed vertical lines represent the 95% confidence interval for the pooled effect.

Fig. 3 illustrates the distribution of effect sizes across all studies. Using an intercept only meta-regression, we found a small-sized effect of risky decision-making task performance on emotion-related impulsivity (ß = 0.086, *t* =5.72, *p* < .0001). Effect size estimates (Fisher’s *z*) from individual studies ranged from −0.48 to 0.60. The proportion of variability in effect sizes from heterogeneity relative to variability from sampling error was moderate to high, *I*^*2*^ = 60.20, which provided evidence for the import of considering moderator effects (Higgins et al., 2019). The between-study variance in effect sizes was small relative to the within-study variances (τ^2^ = 0.0062), which justified the inclusion of a CHE model to test for robustness in the event of a hierarchical covariance structure. When the five studies that reported more than ten effect sizes were removed, the intercept-only model yielded a parallel small and significant effect (ß = 0.088, *t* = 5.53, *p* < .0001).

### 3.3. Moderated meta-regression

**Fig. 4.**
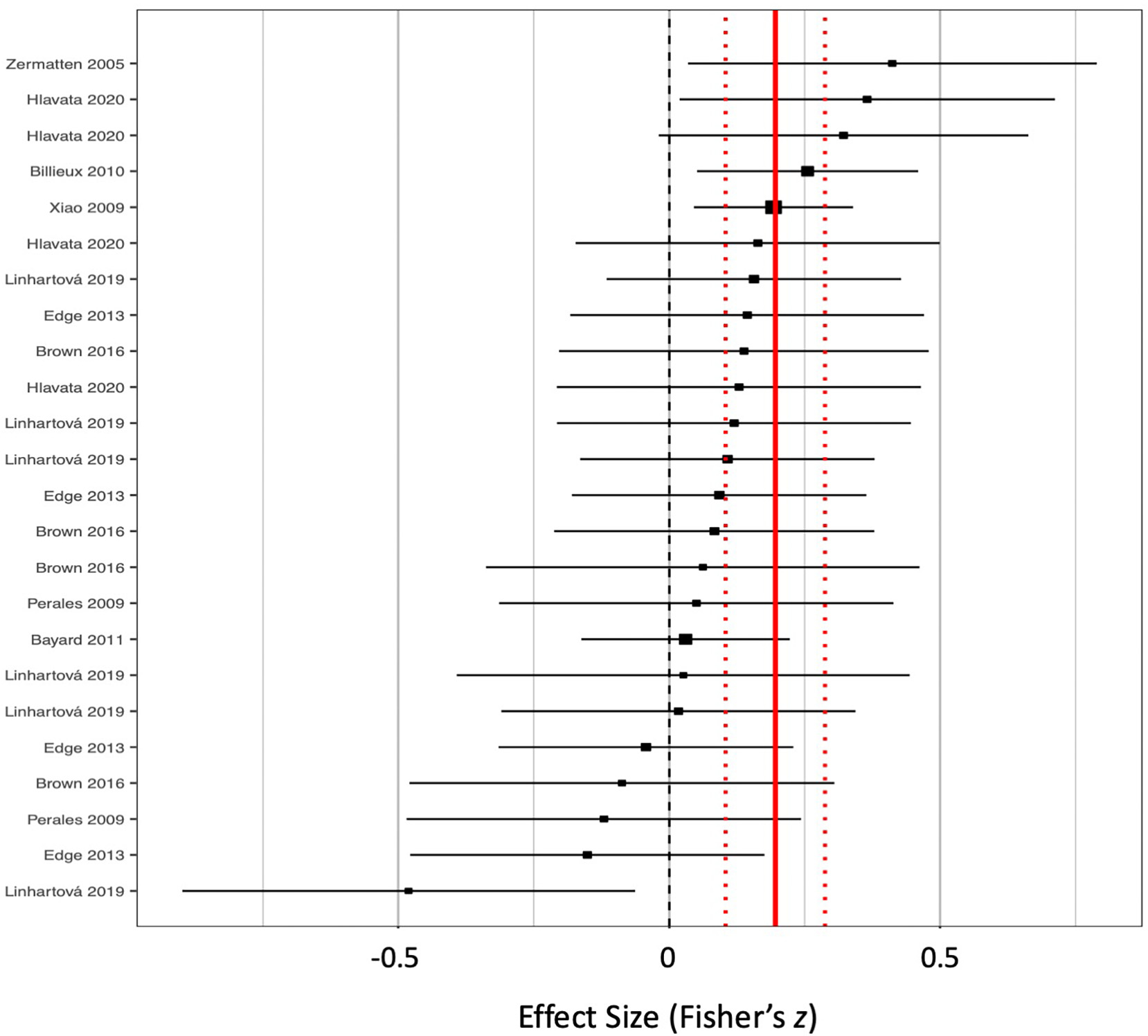
Forest plot for all included effects from the Iowa Gambling Task. Point estimates (squares) with 95% confidence intervals (horizontal lines) are sorted from strongest positive (top) to strongest negative (bottom). Positive effect sizes are those that found high risky decision-making correlating with high emotion-related impulsivity. Solid vertical line represents the pooled effect, estimated by moderated meta-regression. Dashed vertical lines represent the 95% confidence interval for the pooled effect.

**Fig. 5.**
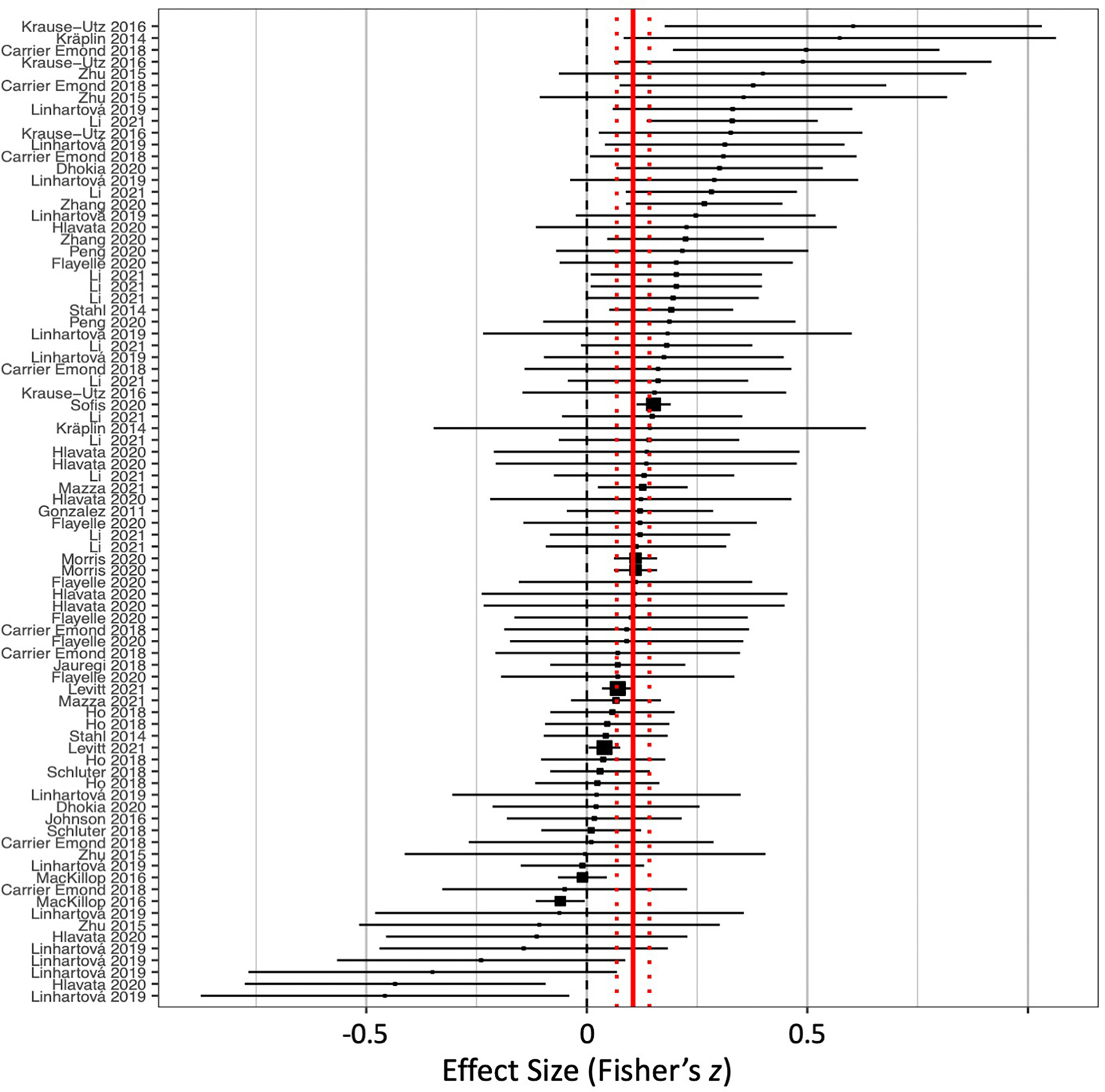
Forest plot for all included effects from the Delay Discounting Task. Point estimates (squares) with 95% confidence intervals (horizontal lines) are sorted from strongest positive (top) to strongest negative (bottom). Positive effect sizes are those that found high risky decision-making correlating with high emotion-related impulsivity. Solid vertical line represents the pooled effect, estimated by moderated meta-regression. Dashed vertical lines represent the 95% confidence interval for the pooled effect.

Table 3 includes model outputs from the full RVE meta-regression with moderators included. The strength of the correlation of risky decision-making with emotion-related impulsivity was moderated significantly by Task Type. In aggregate, scores from the Balloon Analogue Risk Task (ß = 0.050, *t* = 1.71, *p* = 0.114) and Information Sampling Task (ß = 0.016, *t* = 0.29, *p* = 0.783) (Appendices B.2–3), did not correlate significantly with emotion-related impulsivity scores. Scores from the Iowa Gambling Task (ß = 0.197, *t* = 4.26, *p* = 0.011) (Fig. 4), Delay Discounting Task, (ß = 0.108, *t* = 5.78, *p* < 0.001) (Fig. 5), and tasks coded as “other” (ß = 0.145, *t* = 2.98, *p* = 0.017) significantly correlated with emotion-related impulsivity scores.

The Arousal Manipulation moderator showed a nonsignificant trend (ß = 0.109, *t* = 2.38, *p* = 0.051). As shown in Table 3, Monetary Incentive, Sample Type (clinical vs. non-clinical), Measure Type (Negative vs. Positive Urgency scale), Gender, and Age were not significant moderators of effect sizes. In the full model, the proportion of variability in effect sizes from heterogeneity relative to variability from sampling error reduced to a moderate level, *I*^*2*^ = 47.89. The between-study variance was τ^2^ = 0.0047.

Results were parallel when testing higher and lower values of rho (Appendix C.1–2). The Correlated and Hierarchical Effects (CHE) model also had very similar results; however, this version of the model yielded a nonsignificant cumulative effect size for the tasks coded as “Other” (ß = 0.124, 95% CI [−0.003, 0.251]) and a weaker effect of Arousal Manipulation (ß = 0.080, 95% CI [−0.064, 0.223]) (Appendix C.3). When using the re-coded task type variable, the pooled effect was significant for the gambling tasks (ß = 0.158, *t* = 3.84, *p* = 0.003). In this instantiation of the model, the tasks coded as “Other” (ß = 0.180, *t* = 2.63, *p* = 0.050) bordered a significant effect, and the Arousal Manipulation moderator achieved significance (ß = 0.101, *t* = 2.37, *p* = 0.049) – otherwise, the findings remained the same (Appendix C.4).

### 3.4. Closer look at arousal and mood manipulations

Given the relatively small number of studies that experimentally manipulated mood or arousal, none of the unique manipulation types had sufficient power to be examined independently. Despite the limited statistical power, we report effect sizes for different types of mood/arousal inductions separately (Table 2). Studies that induced arousal via stress, imaginal sex, or pharmacology (i.e., yohimbine) reported significant effects within the conditions involving arousal manipulations.

### 3.5. Publication bias

**Fig. 6.**
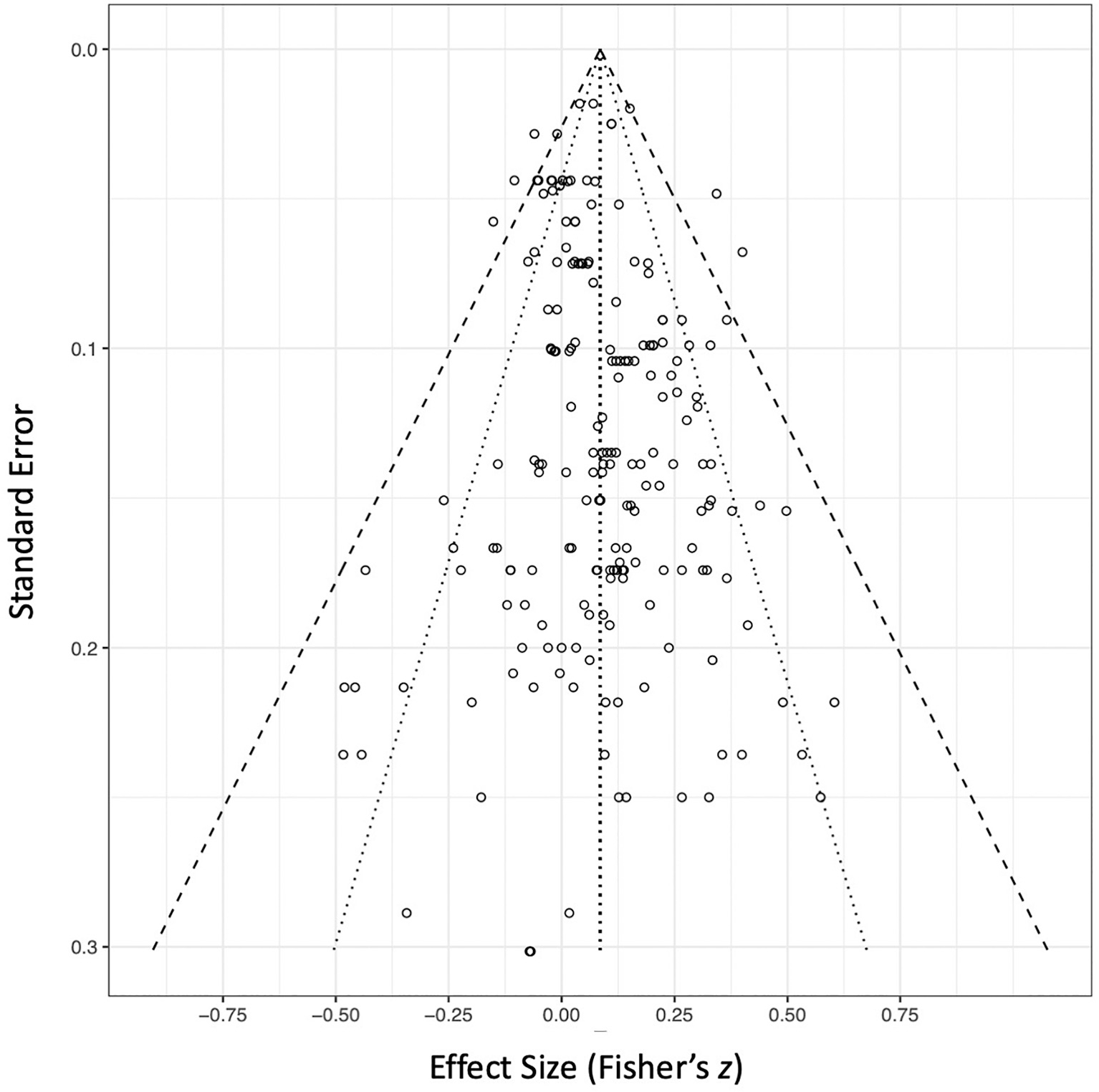
Funnel plot for studies examining effects of risky decision-making and emotion-related impulsivity. Vertical dashed line represents the pooled effect size. Dotted diagonal lines (interior) represent the 95% confidence interval. Dashed diagonal lines (exterior) represent the 99% confidence interval.

To assess publication bias, we first visually inspected a funnel plot of all included effect sizes (Figure 6). Our distribution appeared approximately symmetrical, with small and large sample studies being represented by positive and negative, nonsignificant and significant effects. However, our trim and fill analysis added 30 effect sizes. This indicated a small asymmetry in the funnel plot and justified further evaluation. Our p-curve analysis showed a right-skewed distribution of significant p-values that indicated an absence of systematic “p-hacking” and publication bias (Appendix B.4), such that a true null effect in this literature was highly unlikely (Binomial, *p* = .0007; Continuous (Stouffer), *z* = −6.48, *p* < .0001). Furthermore, the evidential value was determined to not be inadequate or below the 33% power threshold (*z* = 1.89, *p* (one-sided) = .971). The power of the tests included in the p-curve was estimated to be 53% (90% CI: [36%, 68%]. Fourth, our sensitivity analysis concluded that given the 41 affirmative and 154 non-affirmative effect sizes in the dataset, even the most extreme level of publication bias tested (*η* = 200) would not fully attenuate the significant pooled effect (Appendix B.5).

### 3.6. Examination of non-hypothesized sample characteristics and study quality

There was no evidence that non-hypothesized sample characteristics were related to effect sizes (Table 3). The racial identities of sampled participants were only reported for 44.1% of effect sizes. Across those studies that did report on racial identities, there was no effect of non-white race representation on effect size. The year of publication for the included studies ranged from 2005 to 2021, and it was not significantly related to the effect size. 66.2% of effect sizes came from studies that were conducted outside of the United States. There was no significant difference in effect sizes from the United States vs. other countries.

The number of trials was not significantly correlated with effect size across the full set of studies (Table 3). Regarding reporting quality, the percentage of participants identifying as white was reported for 86 of 195 effect sizes. Effects coming from studies that reported on racial identity were on average modestly smaller than effects that came from studies that did not report on race (ß = −0.073, *t* = −2.53, *p* = 0.016). Of the 15 effect sizes from studies that included an arousal manipulation, 9 came from studies that reported information about whether the arousal manipulation succeeded; all reports confirmed success. We did not have enough effect sizes from these studies to conduct a statistical test.

## 4. Discussion

Our systematic review of published and unpublished research on emotion-related impulsivity and risky decision-making uncovered a rich literature with diversity in sample demographics, geographic location, experimental task type, and clinical status. Indeed, beyond synthesizing the published correlations of risky decision-making with emotion-related impulsivity, this study was generative in nature. Over 20 colleagues re-analyzed or shared their raw data, allowing us to add 105 effect sizes to this review that were not previously published, an addition that greatly improved our ability to consider risky decision-making and emotion-related impulsivity effects.

We used a RVE meta-regression approach which provided two core strengths over other meta-analytic approaches. We were able to conserve data from studies that reported more than one relevant effect size, and we were able to examine the relative effects of multiple pre-registered moderator variables concurrently. Across 51 studies and 195 effect sizes of nearly 15,000 participants, we found a small effect between emotion-related impulsivity and increased risk-taking behavior on laboratory decision-making tasks. This effect, though small, was robust. It does not appear that the results were confounded by publication bias. The effect has held steady over the past two decades and generalized to studies conducted outside the United States and across gender and age. Regarding study quality indicators, whether researchers reported participants’ racial identities did relate to effect size, whereas the number of trials in the task did not.

As hypothesized, the meta-correlation between risky decision-making tasks and emotion-related impulsivity was comparable for Positive Urgency and Negative Urgency. This is consistent with recent theory (Johnson et al., 2020) and empirical work showing that Positive Urgency and Negative Urgency are themselves highly correlated and load onto a supraordinate factor (Carver et al., 2011; Cyders and Smith, 2007).

More surprisingly, there was no evidence for moderation by monetary incentives, and the evidence for moderation by arousal manipulation was borderline. As only 15 studies included arousal manipulations, we aggregated the forms of arousal manipulations in the meta-regression. Closer examination of the unique effects indicated that positive mood induction (with perhaps the exception of imaginal sexual rewards) did not strengthen the link between emotion-related impulsivity and risky decision-making. Tentatively, this may reflect that many laboratory positive emotion inductions have small effects on arousal—most participants will not experience large amounts of excitement while completing cognitive tasks in a laboratory setting. Although power was limited, effect sizes from the single studies of stress induction, imaginal sexual rewards, and yohimbine, a pharmacological arousal induction, were significant. These three techniques represent arousal manipulations involving negative valence, positive valence, and without regard to valence respectively. This aligns with the idea that arousal, rather than valence is the component of emotion that intersects with loss of self-control in this type of impulsivity (Johnson et al., 2020). Future work on understanding arousal moderation is of particular importance in this literature.

Contrary to our hypothesis, there was no difference in the aggregate effect sizes of studies that used clinical vs. non-clinical samples. This null effect contrasts a meta-analysis that observed a higher correlation of response inhibition performance with emotion-related impulsivity in clinical samples (*r* = 0.34) than community (*r* = 0.14) and student (*r* = 0.11) samples (Johnson et al., 2016), and with evidence that response inhibition deficits show a curvilinear pattern, in which they are more closely correlated with emotion-related impulsivity within the higher ranges of emotion-related impulsivity (Dekker and Johnson, 2018; Johnson et al., 2016). Tentatively, it may be that neuropsychological correlates are only explanatory at the more severe range of this form of impulsivity, whereas more deliberative decision-making indices could be correlating across a fuller range of emotion-related impulsivity. This idea could be tested more systematically in future research.

Out of the four most common risky decision-making tasks, performance on the Balloon Analogue Risk Task and the Information Sampling Task did not significantly correlate with emotion-related impulsivity. On the other hand, the Iowa Gambling Task, the Delay Discounting Task, and an aggregate of other less common task-types were each correlated with emotion-related impulsivity. Caution is warranted about interpreting the aggregated effect size for this heterogenous set of “other” tasks; however, the data suggest that tasks beyond the four most common are worthy of consideration for future researchers.

Our ability to compare effects by task type shines new light on the cognitive correlates of emotion-related impulsivity. Our findings indicated that the preference for gaining rewards more quickly, as measured on delay discounting tasks, is related to emotion-related impulsivity. Delay discounting tasks provide the purest metrics of decision-making in this literature because they yield isolated time discounting parameters, derived from a set of certain and unambiguous decisions. The other tasks blend decision-making with varying degrees of ambiguity and uncertainty. Among these are the “gambling tasks.” The CGT, Cups Task, GDT, Holt, RGT, and VGT all involve decision-making under conditions of uncertainty, but not ambiguity. That is, the probability contingencies of choices are known, but the outcome of any single trial is unknown. The IGT and CCT are unique among the “gambling tasks” because the probability contingencies of the possible choices are unknown and must be learned implicitly through feedback. Therefore, the IGT and CCT are both uncertain *and* ambiguous. When we included all “gambling tasks” as a single task type, the isolated effect we found for IGT with emotion-related impulsivity remained. From these studies we have evidence that the link of emotion-related impulsivity with risky decision-making impulsivity generalizes across laboratory tasks with varying levels of uncertainty and ambiguity. This generality of the effect across tasks with varying levels of ambiguity and uncertainty may signal that the real-world manifestations of riskier decision-making occur in a wide array of contexts for those with more severe emotion-related impulsivity.

Unlike the DDT or “gambling tasks,” in which participants have an explicit or implicit understanding of the relative risk and reward for making riskier choices, the BART tests risk-taking in a purely stochastic system. This randomness, and consequent lack of available strategy for all participants, may be the ingredient that sets the BART apart from other risky decision-making tasks that do appear to capture some of the variance in emotion-related impulsivity. Our findings appear consistent with findings of one meta-analysis (Sharma et al., 2014) in which a factor analysis showed that the BART did not load onto the same “Impulsive Decision-Making” factor as the IGT and DDT. Although we found evidence that the BART provides a poor laboratory measure of emotion-related impulsivity, there was an absence of BART studies using arousal induction methods that appear most promising (e.g., stress, yohimbine). The Information Sampling Task provides an interesting parallel here. Like the BART, the pooled effect for the IST was nonsignificant, yet in the only study with a pharmacological arousal manipulation, yohimbine administration significantly moderated the relationship between emotion-related impulsivity and risky decision-making on the IST (Herman, Critchley, and Duka, 2019). More work is needed to test whether risky decision-making task performance, including the BART, correlates with emotion-related impulsivity in the context of strong arousal manipulations.

Taken together, we observed a highly significant correlation of emotion-related impulsivity with risky decision-making, which was small but replicated across many types of laboratory tasks. The small aggregate effect size across tasks mirrors a broader issue of poor concordance between trait measures and laboratory experiments of the same construct (Dang, King, and Inzlicht, 2020). One possible explanation is that effect sizes in this literature may be attenuated by the reliability of risky decision-making tasks, as some gambling tasks (i.e., IGT and GDT) have shown low test-retest reliability (Buelow and Barnhart, 2018). This would not appear to be a good explanation for the low effect sizes across tasks, though, as the DDT and the BART have shown acceptable test-retest reliability (Anokhin, Golosheykin, and Mulligan, 2015; Weafer, Baggott, and de Wit, 2013). Beyond reliability, it is possible that tasks and self-ratings capture fundamentally different processes and contexts. For one, there is the necessary methodological disconnect between trait measures which capture “on-average” tendencies across time and “snapshots” of behavioral performance in the laboratory. Together, these factors might lead to an underestimate in the true relationship between the constructs of risky decision-making and emotion-related impulsivity.

### 4.1. Comparison with previous reviews

This meta-regression builds on nearly two decades of research on the laboratory correlates of emotion-related impulsivity, including relevant meta-analyses (Cyders and Coskunpinar, 2011; Sharma et al., 2014). The Cyders and Coskunpinar (2011) review was restricted to examining two studies of laboratory delay discounting, and no other risky decision-making tasks were included. Sharma et al. (2014) expanded this work to include additional risky decision-making tasks, and to differentiate effect sizes by whether rewards were real or hypothetical. The current work extends the findings of these two pioneering meta-analyses by adding 190 new effect sizes, including additional task types, key experimental and sample moderator variables, and greater representation of the Positive Urgency scale, modeled together using recent RVE meta-regression techniques.

### 4.2. Limitations

Despite the strengths, there are several limitations to the present work. First, the generalizability of the findings with regard to racial identity and lifespan development must be interpreted with caution. Our operationalization of race as “white” or “non-white” was chosen to conserve data in the face of different reporting details, but it provided no granularity to examine generalizability across racial identities. Furthermore, most studies did not report race, and those that did report race – which we view as one indicator of the quality of reporting – had smaller effect sizes on average. Also, most samples included only adult participants, so there is insufficient evidence to know if findings generalize across the lifespan. Second, we lacked statistical power to compare some levels of some categorical moderator variables that may exert distinct influences. Third, some moderator variables in this dataset are not distributed evenly across the categories of other moderator variables (e.g., strong arousal manipulations were more common in DDT than BART studies). This is largely accounted for by the structure of the meta-regression; however, it remains formally undifferentiated. Fourth, we did not conduct a forward citation search, and it is possible that we missed one or more relevant findings.

### 4.3. Future directions

Drawing on our findings, we conclude with four discussion points to help guide future work in this area. First, our findings suggest that effects of risky decision-making are present, but modest. Alongside response inhibition, risky decision-making is a cognitive domain that correlates with emotion-related impulsivity, indicating that emotion-related impulsivity is more complex than an isolated cognitive process gone awry (Johnson et al., 2020). It will be important for researchers to move beyond these isolated, small effect sizes to integrate data from multiple cognitive domains using multivariate statistical methods. In this case, we would recommend that researchers use the DDT (unambiguous and certain), RGT (unambiguous and uncertain), and IGT (ambiguous and uncertain) to cover the risky decision-making domain.

Second, when studying emotion-related impulsivity in the lab, we recommend that future researchers carefully select and integrate effective arousal manipulations. The current study demonstrates that monetary incentives and common positive mood inductions do not appear to elicit riskier decisions among people with higher emotion-related impulsivity. However, the few studies using stress inductions, imaginal sex, and pharmacology provide hints that stronger manipulations of physiological arousal may elicit stronger associations between risky decision-making and emotion-related impulsivity. Given the growing body of laboratory studies suggesting that symptoms are evoked after mood inductions for those with this form of impulsivity, and the large body of null findings with “cold” cognitive tasks (Sharma et al., 2014), researchers in this field should effectively manipulate and measure physiological arousal.

Third, our analyses indicate that the correlation between risky decision-making and emotion-related impulsivity generalizes across demographic domains; however, gaps still remain with regard to racial identity and lifespan development. The field will benefit from future studies that recruit racially diverse samples. It will also benefit from increasing the developmental range of inquiry in children, adolescents, and older adults. It is also likely that adversity, oppression, and additional forms of marginalization (e.g., sexual and gender minority and disability status) impact processes relevant for both emotion-related impulsivity (e.g., Carver et al., 2011) and decision-making (e.g., Duffy, McLaughlin, and Green, 2018) and should be evaluated as potential moderators.

Fourth, more research is needed to determine whether increased risky decision-making contributes to poor psychosocial outcomes experienced by individuals with high emotion-related impulsivity. We do not want to label the tendency toward risky decision-making as maladaptive *per se,* especially given the modest size of the effect found here. *Nonetheless*, given that some tasks included in this meta-regression have been linked to real-world reckless driving (Brown et al., 2016), problem gambling (Billieux et al., 2012), risky sexual choices (Derefinko et al., 2014), and binge drinking (Xiao et al., 2009), over time risky decision-making may be connected to the distress and functional impairment experienced by many with high emotion-related impulsivity.

As the current study fills an important gap in the literature on the cognitive correlates of emotion-related impulsivity, so does it highlight the complexity of this critical puzzle. The symptomatic manifestations of emotion-related impulsivity are state-dependent, and most research on risky decision-making has failed to consider this issue. Emotion-related impulsivity also appears to be linked to multiple cognitive domains, with risky decision-making being just one domain implicated. It may even be the case that its cognitive correlates manifest in an equifinal manner. Therefore, it is unlikely that emotion-related impulsivity will be captured by a single index or task even under optimal psychometric and physiological conditions. The findings in this systematic review and meta-regression lead us to believe that risky decision-making tasks will be valuable components of future, multivariate research that will ultimately unlock the neurocognitive code of emotion-related impulsivity, and lead us to more targeted, effective, and transdiagnostic clinical interventions.

## Supplementary Material

supplemental file

## Figures and Tables

**Table 1:** Description of tasks.

Name	Citation	Description

Angling task	Congdon et al., 2013	The participant wins money for catching red fish until they choose to “cash out” or until they catch the one blue fish and go “bankrupt.”
Balloon Analogue Risk Task (BART)	Lejuez et al., 2002	The participant must inflate a balloon to receive more money, but without reaching the point it explodes.
Cambridge Gambling Task (CGT)	Rogers et al., 1999	The participant guesses whether a yellow token is hidden in a red or a blue box out of ten possible boxes.
Columbia Card Task (CCT)	Figner, Mackinlay, Wilkening, and Weber, 2009	Participants sequentially decide how many face down cards to turn over. Win cards add to their winnings, while turning over a loss card subtracts from their total and terminates the trial.
Cups Task	Levin and Hart, 2003	The participant chooses between a safe or a risky option that involves two to five cups of either gains or losses.
Delay Discounting Task (DDT)	Bickel and Marsch, 2001	The participant must choose between an immediate small reward and a delayed larger reward.
Driving simulator task	Brown et al., 2016	The participant drives in highway or urban conditions while his driving behavior measures are assessed.
Game of Dice Task (GDT)	Brand et al., 2005	The participant predicts the outcome of a dice roll by selecting between options with different payoffs.
Holt-Laury Risk Task	Tasoff and Zhang, 2021	Participants choose between high magnitude, low probability rewards and low magnitude, high probability rewards.
Information Sampling Task (IST)	Clark, Robbins, Ersche, and Sahakian, 2006	The participant chooses to obtain more or less information before making a decision.
Iowa Gambling Task (IGT)	Bechara, Tranel, and Damasio, 2000	The participant chooses cards from four decks which either reward or penalize them, while learning that some decks are better than the others.
Obstacle course task	Morrongiello, Stewart, Pope, Pogrebtsova, and Boulay, 2015	A physical risk task where kids must run through a real obstacle course and injury risk behavior is assessed.
Risky Gains Task (RGT)	Kruschwitz, Simmons, Flagan, and Paulus, 2012	The participant can choose a small safe monetary reward or risk losing money for a larger reward.
Single Key Impulsivity Paradigm (SKIP)	Dougherty, Mathias, Marsh, and Jagar, 2005	The participant obtains rewards that are related to the delay between responses.
Two Choice Impulsivity Paradigm (TCIP)	Dougherty et al., 2005	The participant chooses to receive a larger reward accompanied by a longer delay or smaller rewards after a shorter delay.
Verbruggen Gambling Task (VGT)	Verbruggen, Chambers, Lawrence, and McLaren, 2017	Participants choose a small reward that is guaranteed or gamble on larger reward that is uncertain.

**Table 2:** Frequency of categorical moderator variables.

	*N*	*K*	*Pooled ES*

*Sample type*			
Clinical	13	57	0.068
Community	32	101	0.060
Student	16	37	0.101
*Arousal manipulation*			
None	50	180	0.063
Imaginal sex	1	4	0.151
Peer supervision	1	2	0.045
Positive mood	4	5	0.044
Stress	1	2	0.382
Yohimbine	1	2	0.314
*Monetary incentive*			
Yes	9	35	0.003
No	42	160	0.096
ERI measure			
Positive urgency	31	77	0.046
Negative urgency	47	118	0.074
*Task type*			
Angling	1	4	−0.045
BART	13	27	0.026
CGT	2	6	0.160
CCT	1	4	−0.030
Cups	1	1	0.255
DDT	23	83	0.083
Driving	1	16	0.061
GDT	3	3	0.088
Holt	1	2	0.038
IGT	9	24	0.107
IST	4	14	0.028
Obstacle	1	1	0.277
RGT	1	2	0.292
SKIP	1	4	0.063
TCIP	1	2	0.261
VGT	1	2	0.044

*Note:* BART = Balloon Analogue Risk Task, CCT = Columbia Card Task, CGT = Cambridge Gambling Task, DDT = Delay Discounting Task, ERI = Emotion-related impulsivity, GDT = Game of Dice Task, IGT = Iowa Gambling Task, IST = Information Sampling Task, *K* = number of effect sizes, *N* = number of studies, RGT = Risky Gains Task, SKIP = Single Key Impulsivity Paradigm, TCIP = Two Choice Impulsivity Paradigm, VGT = Verbruggen Gambling Task.

**Table 3:** Correlated effects, RVE moderated meta-regression predicting effect size (Fisher’s z), rho = 0.6.

Moderator - level	β	*S.E.*	*t*	df	*p*	95% C.I. Low	95% C.I. high

Task type - BART	0.050	0.029	1.71	11.19	0.1143	−0.014	0.114
Task type - DDT	0.108	0.019	5.78	12.53	0.0001	0.067	0.148
Task type - IGT	0.197	0.046	4.26	4.29	0.0112	0.072	0.322
Task type - IST	0.016	0.054	0.29	4.13	0.7825	−0.132	0.164
Task type - Other	0.145	0.049	2.98	8.39	0.0166	0.034	0.256
Percent female	−0.002	0.019	−0.08	12.64	0.9356	−0.043	0.040
Age	0.025	0.015	1.65	14.22	0.1205	−0.007	0.058
Clinical	−0.040	0.040	−1.01	8.86	0.3388	−0.131	0.050
Arousal	0.109	0.046	2.38	6.65	0.0509	−0.001	0.219
Money	−0.054	0.031	−1.76	10.44	0.1070	−0.122	0.014
ERI measure	−0.020	0.024	−0.81	24.51	0.4280	−0.070	0.031
Percent White^a^	0.006	0.015	0.38	3.74	0.7243	−0.037	0.048
Year^a^	−0.017	0.013	−1.35	17.90	0.1942	−0.044	0.009
Country^a^	−0.047	0.030	−1.59	36.5	0.1207	−0.108	0.013
Reported race^a^	−0.073	0.029	−2.53	36.9	0.0157	−0.131	−0.015
Number of trials^a^	0.020	0.016	1.24	9.74	0.2443	−0.016	0.056

Note:

a = tested separately, BART = Balloon Analogue Risk Task, C.I. = Confidence Interval, DDT = Delay Discounting Task, df = degrees of freedom, ERI = Emotion-related impulsivity, IGT = Iowa Gambling Task, RVE = Robust Variance Estimation, Y/N = Binary variable (i.e., “yes/no”).

## Data Availability

Links to the pre-registration, data, code, and analysis output, which have been posted on the Open Science Framework, are included at the beginning of the Method section within the manuscript.
