# Supplementary material for: Emotion-related impulsivity and risky decision-making: A systematic review and meta-regression
Source: Clin Psychol Rev. Author manuscript; Available in PMC 2024 Mar 1. (PMC9974869; doi:10.1016/j.cpr.2022.102232)
Supplement: supplemental file [file NIHMS1857461-supplement-supplemental_file.docx]

**Appendix A**

*Effect Size and Characteristics of Studies Included in the Meta-Regression (k = 195).*

| Study | *N* | Fisher’ *z* | Task type | ERI Measure | Sample | Age | % female | % white | Country | Arousal | Money | Trials |
| --- | --- | --- | --- | --- | --- | --- | --- | --- | --- | --- | --- | --- |
| Bagge et al., (2013) | 69 | 0.090 | BART | NU | Clinical | 32.19 | 54 | 56 | USA | None | No | 20 |
| Bayard et al., (2011) | 107 | 0.224 | GDT | NU | Community | 28.5 | NA | NA | France | None | No | 18 |
| Bayard et al., (2011) | 107 | 0.030 | IGT | NU | Community | 28.5 | NA | NA | France | None | No | 100 |
| Bellman, (2013)^b^ | 79 | 0.255 | Cups | NU | Student | NA | 40 | 86 | USA | None | No | 54 |
| Billieux et al., (2010) | 95 | 0.255 | IGT | NU | Community | 23.3 | 50.5 | NA | Switzerland | None | No | 100 |
| Booth et al., (2017) | 482 | -0.004 | BART | NU | Community | 13.4 | 55 | 78.9 | UK | None | No | 20 |
| Brown et al., (2016) | 27 | 0.334 | Driving | NU | Clinical | 27.8 | 0 | 85.2 | Canada | None | No | 40 |
| Brown et al., (2016) | 47 | 0.331 | Driving | NU | Community | 30.1 | 0 | 70.2 | Canada | None | No | 40 |
| Brown et al. (2016) | 36 | 0.266 | Driving | NU | Clinical | 30 | 0 | 88.9 | Canada | None | No | 40 |
| Brown et al., (2016) | 28 | 0.237 | Driving | NU | Community | 28.7 | 0 | 53.6 | Canada | None | No | 40 |
| Brown et al., (2016) | 36 | 0.138 | IGT | NU | Clinical | 30 | 0 | 88.9 | Canada | None | No | 100 |
| Brown et al., (2016) | 24 | 0.125 | Driving | NU | Clinical | 27.8 | 0 | 85.2 | Canada | None | No | 40 |
| Brown et al., (2016) | 24 | 0.097 | Driving | NU | Clinical | 27.8 | 0 | 85.2 | Canada | None | No | 40 |
| Brown et al., (2016) | 47 | 0.086 | Driving | NU | Community | 30.1 | 0 | 70.2 | Canada | None | No | 40 |
| Brown et al., (2016) | 47 | 0.083 | IGT | NU | Community | 30.1 | 0 | 70.2 | Canada | None | No | 100 |
| Brown et al., (2016) | 36 | 0.079 | Driving | NU | Clinical | 30 | 0 | 88.9 | Canada | None | No | 40 |
| Brown et al., (2016) | 36 | 0.077 | Driving | NU | Clinical | 30 | 0 | 88.9 | Canada | None | No | 40 |
| Brown et al., (2016) | 27 | 0.062 | IGT | NU | Clinical | 27.8 | 0 | 85.2 | Canada | None | No | 100 |
| Brown et al., (2016) | 47 | 0.055 | Driving | NU | Community | 30.1 | 0 | 70.2 | Canada | None | No | 40 |
| Brown et al., (2016) | 28 | 0.032 | Driving | NU | Community | 28.7 | 0 | 53.6 | Canada | None | No | 40 |
| Brown et al., (2016) | 28 | 0.000 | Driving | NU | Community | 28.7 | 0 | 53.6 | Canada | None | No | 40 |
| Brown et al., (2016) | 28 | -0.030 | Driving | NU | Community | 28.7 | 0 | 53.6 | Canada | None | No | 40 |
| Brown et al., (2016) | 28 | -0.087 | IGT | NU | Community | 28.7 | 0 | 53.6 | Canada | None | No | 100 |
| Brown et al., (2016) | 24 | -0.199 | Driving | NU | Clinical | 27.8 | 0 | 85.2 | Canada | None | No | 40 |
| Brown et al., (2016) | 36 | -0.223 | Driving | NU | Clinical | 30 | 0 | 88.9 | Canada | None | No | 40 |
| Brown et al., (2016) | 47 | -0.261 | Driving | NU | Community | 30.1 | 0 | 70.2 | Canada | None | No | 40 |
| Carrier Emond et al., (2018) | 45 | 0.497 | DDT | NU | Student | 22.77 | 0 | 78.5 | Canada | None | No | 30 |
| Carrier Emond et al., (2018) | 45 | 0.377 | DDT | PU | Student | 22.77 | 0 | 78.5 | Canada | None | No | 30 |
| Carrier Emond et al., (2018) | 45 | 0.310 | DDT | PU | Student | 22.77 | 0 | 78.5 | Canada | Imaginal Sex | No | 30 |
| Carrier Emond et al., (2018) | 45 | 0.161 | DDT | NU | Student | 22.77 | 0 | 78.5 | Canada | Imaginal Sex | No | 30 |
| Carrier Emond et al., (2018) | 53 | 0.090 | DDT | PU | Student | 22.77 | 0 | 78.5 | Canada | Imaginal Sex | No | 30 |
| Carrier Emond et al., (2018) | 53 | 0.070 | DDT | NU | Student | 22.77 | 0 | 78.5 | Canada | Imaginal Sex | No | 30 |
| Carrier Emond et al., (2018) | 53 | 0.010 | DDT | PU | Student | 22.77 | 0 | 78.5 | Canada | None | No | 30 |
| Carrier Emond et al., (2018) | 53 | -0.050 | DDT | NU | Student | 22.77 | 0 | 78.5 | Canada | None | No | 30 |
| Cyders & Coskunpinar, (2012) | 77 | 0.299 | TCIP | NU | Student | 21.1 | 70.1 | NA | USA | None | No | 50 |
| Cyders & Coskunpinar, (2012) | 77 | 0.224 | TCIP | PU | Student | 21.1 | 70.1 | NA | USA | None | No | 50 |
| Cyders, Zapolski et al., (2010) | 102 | 0.107 | BART | PU | Student | 19.22 | 50 | 84 | USA | None | Yes | 30 |
| Cyders, Zapolski et al., (2010) | 102 | -0.023 | BART | PU | Student | 19.22 | 50 | 84 | USA | Pos. Mood^a^ | Yes | 30 |
| Derefinko et al., (2014) | 135 | -0.010 | BART | PU | Student | 19.4 | 0 | NA | USA | None | No | 20 |
| Derefinko et al., (2014) | 135 | -0.030 | BART | NU | Student | 19.4 | 0 | NA | USA | None | No | 20 |
| Dhokia et al., (2020) | 73 | 0.301 | DDT | NU | Community | 45.5 | 65.7 | NA | UK | None | No | 30 |
| Dhokia et al., (2020) | 73 | 0.021 | DDT | PU | Community | 45.5 | 65.7 | NA | UK | None | No | 30 |
| Eben et al., (2020) | 19 | 0.266 | VGT | PU | Student | 21 | 80 | NA | Belgium | None | No | 256 |
| Eben et al., (2020) | 19 | -0.178 | VGT | NU | Student | 21 | 80 | NA | Belgium | None | No | 256 |
| Edge et al., (2013) | 39 | 0.144 | IGT | PU | Community | 33.5 | 59 | NA | USA | None | No | 100 |
| Edge et al., (2013) | 55 | 0.092 | IGT | NU | Clinical | 36 | 65 | NA | USA | None | No | 100 |
| Edge et al., (2013) | 55 | -0.043 | IGT | PU | Clinical | 36 | 65 | NA | USA | None | No | 100 |
| Edge et al., (2013) | 39 | -0.151 | IGT | NU | Community | 33.5 | 59 | NA | USA | None | No | 100 |
| Flayelle et al., (2020) | 58 | 0.203 | DDT | NU | Student | 23.5 | 76.7 | NA | Germany | None | No | 34 |
| Flayelle et al., (2020) | 58 | 0.100 | DDT | NU | Student | 23.5 | 76.7 | NA | Germany | None | No | 34 |
| Flayelle et al., (2020) | 58 | 0.110 | DDT | NU | Student | 23.5 | 76.7 | NA | Germany | None | No | 34 |
| Flayelle et al., (2020) | 58 | 0.070 | DDT | PU | Student | 23.5 | 76.7 | NA | Germany | None | No | 34 |
| Flayelle et al., (2020) | 58 | 0.121 | DDT | PU | Student | 23.5 | 76.7 | NA | Germany | None | No | 34 |
| Flayelle et al., (2020) | 58 | 0.090 | DDT | PU | Student | 23.5 | 76.7 | NA | Germany | None | No | 34 |
| Gonzalez et al., (2011) | 143 | 0.121 | DDT | NU | Student | 21.3 | 69.9 | 71.3 | USA | None | No | 55 |
| Griffin et al., (2018) | 450 | -0.020 | BART | NU | Community | 34.7 | 48 | 84 | USA | None | Yes | 30 |
| Herman et al., (2019) | 21 | 0.532 | IST | PU | Community | 23.19 | 52.3 | NA | UK | Yohimbine^a^ | No | 10 |
| Herman et al., (2019) | 21 | 0.095 | IST | NU | Community | 23.19 | 52.3 | NA | UK | Yohimbine^a^ | No | 10 |
| Herman et al., (2019) | 21 | -0.443 | IST | NU | Community | 21.29 | 57.1 | NA | UK | None | No | 10 |
| Herman et al., (2019) | 21 | -0.483 | IST | PU | Community | 21.29 | 57.1 | NA | UK | None | No | 10 |
| Hlavata et al., (2020) | 35 | 0.365 | IGT | NU | Community | 66.14 | 80.5 | NA | Czech Rep. | None | No | 200 |
| Hlavata et al., (2020) | 36 | 0.322 | IGT | PU | Community | 66.14 | 80.5 | NA | Czech Rep. | None | No | 200 |
| Hlavata et al., (2020) | 36 | 0.226 | DDT | PU | Community | 66.14 | 80.5 | NA | Czech Rep. | None | No | 110 |
| Hlavata et al., (2020) | 37 | 0.163 | IGT | PU | Clinical | 65 | 43.2 | NA | Czech Rep. | None | No | 200 |
| Hlavata et al., (2020) | 35 | 0.136 | DDT | NU | Community | 66.14 | 80.5 | NA | USA | None | No | 110 |
| Hlavata et al., (2020) | 36 | 0.135 | DDT | PU | Community | 66.14 | 80.5 | NA | Czech Rep. | None | No | 110 |
| Hlavata et al., (2020) | 37 | 0.129 | IGT | NU | Clinical | 65 | 43.2 | NA | Czech Rep. | None | No | 200 |
| Hlavata et al., (2020) | 36 | 0.123 | DDT | PU | Clinical | 65 | 43.2 | NA | Czech Rep. | None | No | 110 |
| Hlavata et al., (2020) | 35 | 0.108 | DDT | NU | Community | 66.14 | 80.5 | NA | Czech Rep. | None | No | 110 |
| Hlavata et al., (2020) | 36 | 0.107 | DDT | NU | Clinical | 65 | 43.2 | NA | Czech Rep. | None | No | 110 |
| Hlavata et al., (2020) | 36 | -0.113 | DDT | NU | Clinical | 65 | 43.2 | NA | Czech Rep. | None | No | 110 |
| Hlavata et al., (2020) | 36 | -0.434 | DDT | PU | Clinical | 65 | 43.2 | NA | Czech Rep. | None | No | 110 |
| Ho et al., (2018) | 197 | 0.058 | DDT | NU | Community | 14.7 | 52.3 | 90 | USA | None | Yes | 55 |
| Ho et al., (2018) | 197 | 0.046 | DDT | PU | Community | 14.7 | 52.3 | 90 | USA | None | Yes | 55 |
| Ho et al., (2018) | 197 | 0.037 | DDT | PU | Community | 14.7 | 52.3 | 90 | USA | None | Yes | 55 |
| Ho et al., (2018) | 197 | 0.024 | DDT | NU | Community | 14.7 | 52.3 | 90 | USA | None | Yes | 55 |
| Holfelder et al., (2020) | 86 | 0.126 | GDT | NU | Community | 14 | 50 | NA | Germany | None | No | 18 |
| Jauregi et al., (2018) | 167 | 0.070 | DDT | NU | Student | 19.43 | 85.6 | 40 | UK | None | No | 27 |
| Johnson et al., (2016) | 101 | 0.017 | DDT | PU | Student | 20.82 | 68 | 31 | USA | None | No | 30 |
| Johnson et al., (2016) | 101 | -0.013 | BART | PU | Student | 20.82 | 68 | 31 | USA | Pos. Mood^a^ | No | 31 |
| Johnson et al., (2016) | 101 | -0.016 | BART | PU | Student | 20.82 | 68 | 31 | USA | None | No | 31 |
| Kräplin et al., (2014) | 19 | 0.574 | DDT | NU | Community | 30.63 | 0 | NA | Germany | None | No | 192 |
| Kräplin et al., (2014) | 19 | 0.326 | CGT | NU | Clinical | 31.37 | 0 | NA | Germany | None | No | 72 |
| Kräplin et al., (2014) | 19 | 0.143 | DDT | NU | Clinical | 31.37 | 0 | NA | Germany | None | No | 192 |
| Kräplin et al., (2014) | 19 | 0.127 | CGT | NU | Community | 30.63 | 0 | NA | Germany | None | No | 72 |
| Krause-Utz et al., (2016) | 24 | 0.604 | DDT | NU | Community | 27.53 | 100 | NA | Germany | None | No | 40 |
| Krause-Utz et al., (2016) | 24 | 0.490 | DDT | NU | Community | 27.53 | 100 | NA | Germany | Stress^a^ | No | 40 |
| Krause-Utz et al., (2016) | 46 | 0.326 | DDT | NU | Clinical | 28 | 100 | NA | Germany | Stress^a^ | No | 40 |
| Krause-Utz et al., (2016) | 46 | 0.153 | DDT | NU | Clinical | 28 | 100 | NA | Germany | None | No | 40 |
| Kvam et al., (2020) | 220 | 0.400 | CGT | NU | Community | 26.6 | 40 | NA | USA | None | No | 72 |
| Kvam et al., (2020) | 431 | 0.343 | CGT | NU | Clinical | 29.2 | 25 | NA | USA | None | No | 72 |
| Kvam et al., (2020) | 431 | -0.040 | CGT | PU | Clinical | 29.2 | 25 | NA | USA | None | No | 72 |
| Kvam et al., (2020) | 220 | -0.060 | CGT | PU | Community | 26.6 | 40 | NA | USA | None | No | 72 |
| Levitt et al., (2021) | 3021 | 0.07 | DDT | NU | Community | 35.3 | 56.7 | 70.2 | USA | None | No | 5 |
| Levitt et al., (2021) | 3021 | 0.04 | DDT | NU | Community | 35.3 | 56.7 | 70.2 | USA | None | No | 5 |
| Li et al., (2021) | 103 | 0.021 | BART | PU | Clinical | 50 | 27.8 | NA | China | None | No | 20 |
| Li et al., (2021) | 105 | 0.195 | DDT | PU | Clinical | 50 | 27.8 | NA | China | None | No | 9 |
| Li et al., (2021) | 105 | 0.181 | DDT | PU | Clinical | 50 | 27.8 | NA | China | None | No | 9 |
| Li et al., (2021) | 105 | 0.329 | DDT | PU | Clinical | 50 | 27.8 | NA | China | None | No | 9 |
| Li et al., (2021) | 103 | -0.024 | BART | NU | Clinical | 50 | 27.8 | NA | China | None | No | 20 |
| Li et al., (2021) | 105 | 0.203 | DDT | NU | Clinical | 50 | 27.8 | NA | China | None | No | 9 |
| Li et al., (2021) | 105 | 0.203 | DDT | NU | Clinical | 50 | 27.8 | NA | China | None | No | 9 |
| Li et al., (2021) | 105 | 0.282 | DDT | NU | Clinical | 50 | 27.8 | NA | China | None | No | 9 |
| Li et al., (2021) | 87 | 0.198 | BART | PU | Community | 48.2 | 33.9 | NA | China | None | No | 20 |
| Li et al., (2021) | 95 | 0.148 | DDT | PU | Community | 48.2 | 33.9 | NA | China | None | No | 9 |
| Li et al., (2021) | 95 | 0.161 | DDT | PU | Community | 48.2 | 33.9 | NA | China | None | No | 9 |
| Li et al., (2021) | 95 | 0.130 | DDT | PU | Community | 48.2 | 33.9 | NA | China | None | No | 9 |
| Li et al., (2021) | 87 | 0.243 | BART | NU | Community | 48.2 | 33.9 | NA | China | None | No | 20 |
| Li et al., (2021) | 95 | 0.141 | DDT | NU | Community | 48.2 | 33.9 | NA | China | None | No | 9 |
| Li et al., (2021) | 95 | 0.111 | DDT | NU | Community | 48.2 | 33.9 | NA | China | None | No | 9 |
| Li et al., (2021) | 95 | 0.121 | DDT | NU | Community | 48.2 | 33.9 | NA | China | None | No | 9 |
| Linhartová, Sirucek et al., (2019) | 200 | -0.010 | DDT | NU | Student | 23.04 | 54 | NA | Czech Rep. | None | No | 110 |
| Linhartová, Latalova, et al., (2019) | 55 | 0.331 | DDT | NU | Community | 23.42 | 63.63 | NA | Czech Rep. | None | No | 110 |
| Linhartová, Latalova, et al., (2019) | 55 | 0.313 | DDT | NU | Community | 23.42 | 63.63 | NA | Czech Rep. | None | No | 110 |
| Linhartová, Latalova, et al., (2019) | 39 | 0.289 | DDT | NU | Clinical | 23.39 | 87.17 | NA | Czech Rep. | None | No | 110 |
| Linhartová, Latalova, et al., (2019) | 55 | 0.247 | DDT | PU | Community | 23.42 | 63.63 | NA | Czech Rep. | None | No | 110 |
| Linhartová, Latalova, et al., (2019) | 25 | 0.183 | DDT | PU | Clinical | 23.28 | 24 | NA | Czech Rep. | None | No | 110 |
| Linhartová, Latalova, et al., (2019) | 55 | 0.175 | DDT | PU | Community | 23.42 | 63.63 | NA | Czech Rep. | None | No | 110 |
| Linhartová, Latalova, et al., (2019) | 55 | 0.156 | IGT | NU | Community | 23.42 | 63.63 | NA | Czech Rep. | None | No | 200 |
| Linhartová, Latalova, et al., (2019) | 39 | 0.120 | IGT | NU | Clinical | 23.39 | 87.17 | NA | Czech Rep. | None | No | 200 |
| Linhartová, Latalova, et al., (2019) | 55 | 0.107 | IGT | PU | Community | 23.42 | 63.63 | NA | Czech Rep. | None | No | 200 |
| Linhartová, Latalova, et al., (2019) | 25 | 0.026 | IGT | PU | Clinical | 23.28 | 24 | NA | Czech Rep. | None | No | 200 |
| Linhartová, Latalova, et al., (2019) | 39 | 0.022 | DDT | NU | Clinical | 23.39 | 87.17 | NA | Czech Rep. | None | No | 110 |
| Linhartová, Latalova, et al., (2019) | 39 | 0.017 | IGT | PU | Clinical | 23.39 | 87.17 | NA | Czech Rep. | None | No | 200 |
| Linhartová, Latalova, et al., (2019) | 25 | -0.062 | DDT | PU | Clinical | 23.28 | 24 | NA | Czech Rep. | None | No | 110 |
| Linhartová, Latalova, et al., (2019) | 39 | -0.143 | DDT | PU | Clinical | 23.39 | 87.17 | NA | Czech Rep. | None | No | 110 |
| Linhartová, Latalova, et al., (2019) | 39 | -0.239 | DDT | PU | Clinical | 23.39 | 87.17 | NA | Czech Rep. | None | No | 110 |
| Linhartová, Latalova, et al., (2019) | 25 | -0.350 | DDT | NU | Clinical | 23.28 | 24 | NA | Czech Rep. | None | No | 110 |
| Linhartová, Latalova, et al., (2019) | 25 | -0.457 | DDT | NU | Clinical | 23.28 | 24 | NA | Czech Rep. | None | No | 110 |
| Linhartová, Latalova, et al., (2019) | 25 | -0.481 | IGT | NU | Clinical | 23.28 | 24 | NA | Czech Rep. | None | No | 200 |
| Mackillop et al., (2016) | 1252 | -0.010 | DDT | NU | Community | 21.5 | 62.2 | 83.5 | USA | None | Yes | 80 |
| Mackillop et al., (2016) | 1252 | -0.060 | DDT | PU | Community | 21.5 | 62.2 | 83.5 | USA | None | Yes | 80 |
| Mazza et al., (2021) | 522 | -0.023 | Angling | PU | Community | 33.6 | 50.2 | 78.8 | USA | None | Yes | 30 |
| Mazza et al., (2021) | 522 | -0.054 | Angling | PU | Community | 33.6 | 50.2 | 78.8 | USA | None | Yes | 30 |
| Mazza et al., (2021) | 522 | -0.053 | Angling | NU | Community | 33.6 | 50.2 | 78.8 | USA | None | Yes | 30 |
| Mazza et al., (2021) | 522 | -0.051 | Angling | NU | Community | 33.6 | 50.2 | 78.8 | USA | None | Yes | 30 |
| Mazza et al., (2021) | 522 | -0.104 | Columbia | NU | Community | 33.6 | 50.2 | 78.8 | USA | None | Yes | 54 |
| Mazza et al., (2021) | 522 | -0.021 | Columbia | NU | Community | 33.6 | 50.2 | 78.8 | USA | None | Yes | 54 |
| Mazza et al., (2021) | 522 | 0.002 | Columbia | PU | Community | 33.6 | 50.2 | 78.8 | USA | None | Yes | 54 |
| Mazza et al., (2021) | 522 | 0.002 | Columbia | PU | Community | 33.6 | 50.2 | 78.8 | USA | None | Yes | 54 |
| Mazza et al., (2021) | 374 | 0.127 | DDT | PU | Community | 33.6 | 50.2 | 78.8 | USA | None | Yes | 11 |
| Mazza et al., (2021) | 374 | 0.066 | DDT | NU | Community | 33.6 | 50.2 | 78.8 | USA | None | Yes | 11 |
| Mazza et al., (2021) | 522 | 0.020 | Holt | NU | Community | 33.6 | 50.2 | 78.8 | USA | None | Yes | 10 |
| Mazza et al., (2021) | 522 | 0.056 | Holt | PU | Community | 33.6 | 50.2 | 78.8 | USA | None | Yes | 10 |
| Mazza et al., (2021) | 514 | 0.074 | IST | PU | Community | 33.6 | 50.2 | 78.8 | USA | None | Yes | 20 |
| Mazza et al., (2021) | 514 | 0.014 | IST | NU | Community | 33.6 | 50.2 | 78.8 | USA | None | Yes | 20 |
| Millner et al., (2020) | 30 | 0.106 | IST | NU | Clinical | 26.8 | 60 | 83.3 | USA | None | No | 10 |
| Millner et al., (2020) | 30 | -0.043 | IST | PU | Clinical | 26.8 | 60 | 83.3 | USA | None | No | 10 |
| Millner et al., (2020) | 31 | 0.092 | IST | NU | Clinical | 26.2 | 61.3 | 74.2 | USA | None | No | 10 |
| Millner et al., (2020) | 31 | 0.061 | IST | PU | Clinical | 26.2 | 61.3 | 74.2 | USA | None | No | 10 |
| Millner et al., (2020) | 32 | 0.195 | IST | NU | Community | 27.2 | 65.6 | 76.5 | USA | None | No | 10 |
| Millner et al., (2020) | 32 | -0.081 | IST | PU | Community | 27.2 | 65.6 | 76.5 | USA | None | No | 10 |
| Moreno-Padilla et al., (2018) | 56 | -0.060 | BART | PU | Community | 15.35 | 57 | NA | Spain | None | No | 20 |
| Morris et al., (2020) | 1609 | 0.110 | DDT | NU | Community | 37 | 59.6 | 72.8 | USA | None | No | 10 |
| Morris et al., (2020) | 1609 | 0.110 | DDT | NU | Community | 37 | 59.6 | 72.8 | USA | None | No | 10 |
| Morrongiello et al., (2015) | 68 | 0.277 | Obstacle | PU | Community | 8.05 | 53 | 99 | Canada | Pos. Mood^a^ | No | NA |
| Nuyens et al., (2016) | 36 | 0.313 | SKIP | PU | Community | 21.35 | 16.03 | NA | Belgium | None | Yes | 4 |
| Nuyens et al., (2016) | 36 | 0.116 | SKIP | NU | Community | 21.35 | 16.03 | NA | Belgium | None | Yes | 4 |
| Nuyens et al., (2016) | 36 | -0.065 | SKIP | NU | Community | 21.35 | 16.03 | NA | Belgium | None | Yes | 4 |
| Nuyens et al., (2016) | 36 | -0.112 | SKIP | PU | Community | 21.35 | 16.03 | NA | Belgium | None | Yes | 4 |
| Peng et al., (2020) | 50 | 0.187 | DDT | PU | Student | 21.5 | 68.8 | NA | China | None | Yes | 256 |
| Peng et al., (2020) | 50 | 0.216 | DDT | NU | Student | 21.5 | 68.8 | NA | China | None | Yes | 256 |
| Perales et al., (2009) | 32 | 0.050 | IGT | PU | Student | 21.5 | 100 | NA | Spain | None | No | 100 |
| Perales et al., (2009) | 32 | -0.121 | IGT | NU | Student | 21.5 | 100 | NA | Spain | None | No | 100 |
| Reniers et al., (2017) | 201 | 0.161 | BART | NU | Community | 19.8 | 79 | 78.6 | UK | None | Yes | 30 |
| Reniers et al., (2017) | 201 | 0.060 | BART | NU | Community | 19.8 | 79 | 78.6 | UK | Peer Sup. | Yes | 30 |
| Reniers et al., (2017) | 201 | 0.029 | BART | PU | Community | 19.8 | 79 | 78.6 | UK | Peer Sup. | Yes | 30 |
| Reniers et al., (2017) | 201 | -0.074 | BART | PU | Community | 19.8 | 79 | 78.6 | UK | None | Yes | 30 |
| Schluter et al., (2018) | 304 | 0.030 | BART | PU | Clinical | 37.57 | 45 | 80.3 | USA | None | No | 30 |
| Schluter et al., (2018) | 304 | 0.030 | DDT | PU | Clinical | 37.57 | 45 | 80.3 | USA | None | No | 77 |
| Schluter et al., (2018) | 304 | 0.010 | DDT | NU | Clinical | 37.57 | 45 | 80.3 | USA | None | No | 77 |
| Schluter et al., (2018) | 304 | -0.151 | BART | NU | Clinical | 37.57 | 45 | 80.3 | USA | None | No | 30 |
| Snorrason et al., (2011) | 55 | -0.141 | IST | NU | Clinical | 26.3 | 92.7 | 100 | Iceland | None | No | 10 |
| Snorrason et al., (2011) | 55 | -0.050 | IST | PU | Clinical | 26.3 | 92.7 | 100 | Iceland | None | No | 10 |
| Sofis et al., (2020) | 2545 | 0.151 | DDT | NU | Community | 48.4 | 40.4 | 88.5 | USA | None | No | 5 |
| Squillace et al., (2019) | 230 | 0.010 | GDT | NU | Community | 28.4 | 65 | NA | Argentina | None | No | 30 |
| Stahl et al., (2014) | 198 | 0.191 | DDT | NU | Student | 25.46 | 65.6 | NA | Germany | None | No | 7 |
| Stahl et al., (2014) | 198 | 0.043 | DDT | NU | Student | 25.46 | 65.6 | NA | Germany | None | No | 7 |
| Steward et al., (2019) | 46 | 0.439 | RGT | PU | Community | 33.57 | 100 | NA | Spain, Aust. | None | Yes | 96 |
| Steward et al., (2019) | 46 | 0.145 | RGT | NU | Community | 33.57 | 100 | NA | Spain, Aust. | None | Yes | 96 |
| Um et al., (2021)^b^ | 15 | 0.017 | BART | NU | Community | 31.33 | 26.7 | 66.7 | USA | None | No | 30 |
| Um et al., (2021)^b^ | 14 | -0.068 | BART | NU | Community | 29.21 | 28.6 | 78.6 | USA | Pos. Mood^a^ | No | 30 |
| Um et al., (2021)^b^ | 14 | -0.071 | BART | PU | Community | 29.21 | 28.6 | 78.6 | USA | Pos. Mood^a^ | No | 30 |
| Um et al., (2021)^b^ | 15 | -0.33 | BART | PU | Community | 31.33 | 26.7 | 66.7 | USA | None | No | 30 |
| Xiao et al., (2009) | 181 | 0.192 | IGT | NU | Student | 16.2 | 54.6 | NA | USA, China | None | No | 100 |
| Yau et al., (2015) | 66 | 0.080 | BART | NU | Community | 14.65 | 54 | 69.7 | USA | None | No | 60 |
| Zermatten et al., (2005) | 30 | 0.412 | IGT | NU | Student | 23.3 | 50 | NA | Switzerland | None | No | 100 |
| Zhang et al., (2020) | 125 | 0.35 | BART | NU | Community | 46 | 37 | NA | China | None | No | 20 |
| Zhang et al., (2020) | 125 | 0.26 | DDT | PU | Community | 46 | 37 | NA | China | None | No | 27 |
| Zhang et al., (2020) | 125 | 0.22 | DDT | NU | Community | 46 | 37 | NA | China | None | No | 27 |
| Zhang et al., (2020) | 125 | 0.22 | BART | PU | Community | 46 | 37 | NA | China | None | No | 20 |
| Zhu et al., (2017) | 21 | 0.399 | DDT | NU | Clinical | 31.9 | 32 | NA | USA | None | No | 137 |
| Zhu et al., (2017) | 21 | 0.355 | DDT | PU | Clinical | 31.9 | 32 | NA | USA | None | No | 137 |
| Zhu et al., (2017) | 26 | -0.004 | DDT | NU | Community | 26.3 | 57.7 | NA | USA | None | No | 137 |
| Zhu et al., (2017) | 26 | -0.107 | DDT | PU | Community | 26.3 | 57.7 | NA | USA | None | No | 137 |

*Note*: A data file with additional details is available as part of the Open Science Framework registration for this study. BART = Balloon Analogue Risk Task, CGT = Cambridge Gambling Task, DDT = Delay Discounting Task, ERI = Emotion-related impulsivity, GDT = Game of Dice Task, IGT = Iowa Gambling Task, IST = Information Sampling Task, *N* = sample size, NA = data missing, NU = Negative Urgency, PU = Positive Urgency, RGT = Risky Gains Task, SKIP = Single Key Impulsivity Paradigm, TCIP = Two Choice Impulsivity Paradigm, VGT = Verbruggen Gambling Task

^a^Checked that arousal manipulation was successful. ^b^Effect size extracted from a study that has not been published.

**Appendix B** – we include five supplemental figures referenced in the manuscript.

**Supplemental Figure B.1 -** Distribution of the number of effect sizes reported per study.

**Supplemental Figure B.2 -** Forest plot for all included effects from the Balloon Analogue Risk Task. Point estimates (squares) with 95% confidence intervals (horizontal lines) are sorted from strongest positive (top) to strongest negative (bottom). Positive effect sizes are those that found high risky decision-making correlating with high emotion-related impulsivity. Solid red line represents the pooled effect, estimated by moderated meta-regression. Dashed red lines represent the 95% confidence interval for the pooled effect.

Effect Size (Fisher’s *z*)

**Supplemental Figure B.3 -** Forest plot for all included effects from the Information Sampling Task. Point estimates (squares) with 95% confidence intervals (horizontal lines) are sorted from strongest positive (top) to strongest negative (bottom). Positive effect sizes are those that found high risky decision-making correlating with high emotion-related impulsivity. Solid red line represents the pooled effect, estimated by moderated meta-regression. Dashed red lines represent the 95% confidence interval for the pooled effect.

Effect Size (Fisher’s *z*)

**Supplemental Figure B.4 -** P-curve plot. Blue line shows the distribution of significant *p* values (*k* = 49) across the included effect sizes. The red dotted line shows the expected distribution of significant *p* values if there is no true effect. The green dashed line shows the expected distribution of significant *p* values with 33% statistical power. The observed curve is right skewed – 36 out of 49 effects are *p* < .025 – which indicates that there is evidential value for the relationship between behavioral indices of risky decision-making and emotion-related impulsivity.

**Supplemental Figure B.5 -** Sensitivity analysis examining the attenuation of the common effect estimate based on the severity of simulated publication bias. $\eta=$ the number of times more likely an affirmative study is to be published than a non-affirmative study. Gray shading represents the 95% confidence interval.

**Appendix C** – in the interest of transparency, we have included model coefficients, statistics, and confidence intervals for four meta-regression models built to test the robustness of our primary meta-regression. Supplemental Tables 1a and 1b show models that test varying levels of rho (the assumed within-sample correlation). Supplemental Table 2 shows a model that tests a Correlated and Hierarchical Effects model, and Supplemental Table 3 shows a model that tests a second way of coding task type.

**Supplemental Table C.1**

*Correlated Effects, RVE Moderated Meta-Regression Predicting Effect Size (Fisher’s z) with rho = 0.4*

| Moderator - Level | Estimate (ß) | Standard Error | *t*-value | df | *p*-value | 95% C.I. Low | 95% C.I. High |
| --- | --- | --- | --- | --- | --- | --- | --- |
| Task type - BART | 0.050 | 0.029 | 1.72 | 11.15 | 0.1138 | -0.014 | 0.114 |
| Task type - DDT | 0.108 | 0.019 | 5.78 | 12.49 | 0.0001 | 0.067 | 0.148 |
| Task type - IGT | 0.197 | 0.046 | 4.28 | 4.28 | 0.0112 | 0.072 | 0.322 |
| Task type - IST | 0.016 | 0.054 | 0.30 | 4.13 | 0.7793 | -0.131 | 0.164 |
| Task type - Other | 0.145 | 0.049 | 2.98 | 8.36 | 0.0168 | 0.034 | 0.256 |
| % Female | -0.002 | 0.019 | -0.09 | 12.63 | 0.9335 | -0.043 | 0.040 |
| Age | 0.025 | 0.015 | 1.65 | 14.21 | 0.1199 | -0.007 | 0.058 |
| Clinical | -0.040 | 0.040 | -1.01 | 8.82 | 0.3386 | -0.131 | 0.050 |
| Arousal | 0.109 | 0.046 | 2.38 | 6.64 | 0.0509 | -0.001 | 0.219 |
| Money | -0.054 | 0.031 | -1.77 | 10.40 | 0.1059 | -0.122 | 0.014 |
| ERI Measure | -0.020 | 0.024 | -0.80 | 24.39 | 0.4289 | -0.070 | 0.031 |

*Note*: BART = Balloon Analogue Risk Task, C.I. = Confidence Interval, DDT = Delay Discounting Task, df = degrees of freedom, ERI = Emotion-related impulsivity, IGT = Iowa Gambling Task, IST = Information Sampling Task, RVE = Robust Variance Estimation, Y/N = Binary variable (i.e., “yes/no”)

**Supplemental Table C.2**

*Correlated Effects, RVE Moderated Meta-Regression Predicting Effect Size (Fisher’s z), with rho = 0.8*

| Moderator - Level | Estimate (ß) | Standard Error | *t*-value | df | *p*-value | 95% C.I. Low | 95% C.I. High |
| --- | --- | --- | --- | --- | --- | --- | --- |
| Task type - BART | 0.050 | 0.029 | 1.71 | 11.23 | 0.1148 | -0.014 | 0.114 |
| Task type - DDT | 0.108 | 0.019 | 5.78 | 12.58 | 0.0001 | 0.067 | 0.149 |
| Task type - IGT | 0.197 | 0.046 | 4.26 | 4.30 | 0.0112 | 0.072 | 0.322 |
| Task type - IST | 0.016 | 0.054 | 0.29 | 4.14 | 0.7857 | -0.132 | 0.163 |
| Task type - Other | 0.145 | 0.049 | 2.99 | 8.42 | 0.0164 | 0.034 | 0.257 |
| % Female | -0.002 | 0.019 | -0.08 | 12.65 | 0.9377 | -0.043 | 0.040 |
| Age | 0.025 | 0.015 | 1.65 | 14.23 | 0.1210 | -0.008 | 0.058 |
| Clinical | -0.040 | 0.040 | -1.01 | 8.91 | 0.3389 | -0.131 | 0.050 |
| Arousal | 0.109 | 0.046 | 2.38 | 6.66 | 0.0510 | -0.001 | 0.219 |
| Money | -0.054 | 0.031 | -1.76 | 10.46 | 0.1080 | -0.122 | 0.014 |
| ERI Measure | -0.020 | 0.024 | -0.81 | 24.63 | 0.4271 | -0.070 | 0.031 |

*Note*: BART = Balloon Analogue Risk Task, C.I. = Confidence Interval, DDT = Delay Discounting Task, df = degrees of freedom, ERI = Emotion-related impulsivity, IGT = Iowa Gambling Task, IST = Information Sampling Task, RVE = Robust Variance Estimation, Y/N = Binary variable (i.e., “yes/no”)

**Supplemental Table C.3**

*Correlated & Hierarchical Effects, RVE Moderated Meta-Regression Predicting Effect Size (Fisher’s z), with rho = 0.6*

| Moderator - Level | Estimate (ß) | Standard Error | df | 95% C.I. Low | 95% C.I. High |
| --- | --- | --- | --- | --- | --- |
| Task type - BART | 0.066 | 0.034 | 11.29 | -0.008 | 0.140 |
| Task type - DDT | 0.121 | 0.022 | 19.59 | 0.075 | 0.167 |
| Task type - IGT | 0.157 | 0.050 | 6.58 | 0.038 | 0.276 |
| Task type - IST | 0.077 | 0.064 | 2.64 | -0.143 | 0.298 |
| Task type - Other | 0.124 | 0.056 | 8.83 | -0.003 | 0.251 |
| % Female | 0.023 | 0.024 | 9.19 | -0.030 | 0.077 |
| Age | 0.031 | 0.019 | 13.30 | -0.010 | 0.072 |
| Clinical Y/N | -0.065 | 0.045 | 8.56 | -0.168 | 0.038 |
| Arousal | 0.080 | 0.058 | 5.88 | -0.064 | 0.223 |
| Money | -0.077 | 0.034 | 9.72 | -0.154 | -0.000 |
| ERI Measure | -0.009 | 0.020 | 16.53 | -0.050 | 0.033 |

*Note*: BART = Balloon Analogue Risk Task, C.I. = Confidence Interval, DDT = Delay Discounting Task, df = degrees of freedom, ERI = Emotion-related impulsivity, IGT = Iowa Gambling Task, RVE = Robust Variance Estimation, Y/N = Binary variable (i.e., “yes/no”)

**Supplemental Table C.4**

*Correlated Effects, RVE Moderated Meta-Regression Predicting Effect Size (Fisher’s z) with rho = 0.6*

| Moderator - Level | Estimate (ß) | Standard Error | *t*-value | df | *p*-value | 95% C.I. Low | 95% C.I. High |
| --- | --- | --- | --- | --- | --- | --- | --- |
| Task type - BART | 0.053 | 0.029 | 1.84 | 11.26 | 0.0916 | -0.010 | 0.117 |
| Task type - DDT | 0.109 | 0.019 | 5.76 | 12.60 | 0.0001 | 0.068 | 0.150 |
| Task type - Gambling | 0.158 | 0.041 | 3.84 | 10.43 | 0.0030 | 0.067 | 0.249 |
| Task type - IST | 0.016 | 0.055 | 0.29 | 4.15 | 0.7835 | -0.133 | 0.165 |
| Task type - Other | 0.180 | 0.068 | 2.63 | 4.63 | 0.0501 | -0.000 | 0.360 |
| % Female | 0.001 | 0.018 | 0.07 | 12.54 | 0.9431 | -0.038 | 0.040 |
| Age | 0.025 | 0.016 | 1.59 | 14.17 | 0.1346 | -0.009 | 0.058 |
| Clinical | -0.040 | 0.039 | -1.02 | 8.95 | 0.3331 | -0.129 | 0.049 |
| Arousal | 0.101 | 0.043 | 2.37 | 7.09 | 0.0491 | 0.001 | 0.202 |
| Money | -0.059 | 0.031 | -1.89 | 10.44 | 0.0876 | -0.128 | 0.010 |
| ERI Measure | -0.023 | 0.024 | -0.94 | 24.29 | 0.3561 | -0.072 | 0.027 |

*Note*: BART = Balloon Analogue Risk Task, C.I. = Confidence Interval, DDT = Delay Discounting Task, df = degrees of freedom, ERI = Emotion-related impulsivity, RVE = Robust Variance Estimation, Y/N = Binary variable (i.e., “yes/no”)

**Appendix D** – Here we list the citations for the studies that yielded data used in this meta-regression but were not cited in the manuscript.

*Bagge, C. L., Littlefield, A. K., Rosellini, A. J., & Coffey, S. F. (2013). Relations among behavioral and questionnaire measures of impulsivity in a sample of suicide attempters. *Suicide and Life-Threatening Behavior*, *43*(4), 460–467. <http://dx.doi.org.libproxy.berkeley.edu/10.1111/sltb.12030>

*Bayard, S., Raffard, S., & Gely-Nargeot, M.C. (2011). Do facets of self-reported impulsivity predict decision-making under ambiguity and risk? Evidence from a community sample. *Psychiatry Research*, *190*(2–3), 322–326. <http://dx.doi.org.libproxy.berkeley.edu/10.1016/j.psychres.2011.06.013>

*Bellman, S. B. (2013). I would rather be happy than right: Consumer impulsivity, risky decision making, and accountability [The University of Iowa]. In *Dissertation Abstracts International Section A: Humanities and Social Sciences* (Vol. 73, Issues 11-A(E), p. No Pagination Specified). <http://search.proquest.com/psycinfo/docview/1373445471/EB9107C7E4924D2CPQ/24>

*Billieux, J., Gay, P., Rochat, L., & Van der Linden, M. (2010). The role of urgency and its underlying psychological mechanisms in problematic behaviours. *Behaviour Research and Therapy*, *48*(11), 1085–1096. <http://dx.doi.org.libproxy.berkeley.edu/10.1016/j.brat.2010.07.008>

*Booth, C., Songco, A., Parsons, S., Heathcote, L., Vincent, J., Keers, R., & Fox, E. (2017). The CogBIAS longitudinal study protocol: Cognitive and genetic factors influencing psychological functioning in adolescence. *BMC Psychology*, *5*(1), 1–14. <http://dx.doi.org.libproxy.berkeley.edu/10.1186/s40359-017-0210-3>

*Brown, T. G., Ouimet, M. C., Eldeb, M., Tremblay, J., Vingilis, E., Nadeau, L., Pruessner, J., & Bechara, A. (2016). Personality, executive control, and neurobiological characteristics associated with different forms of risky driving. *PLoS ONE*, *11*(2), e0150227. <https://doi.org/10.1371/journal.pone.0150227>

*Carrier Emond, F., Gagnon, J., Nolet, K., Cyr, G., & Rouleau, J. L. (2018). What money can’t buy: Different patterns in decision making about sex and money predict past sexual coercion perpetration. *Archives of Sexual Behavior*, *47*(2), 429–441. <http://dx.doi.org.libproxy.berkeley.edu/10.1007/s10508-017-1116-0>

*Cyders, M. A., & Coskunpinar, A. (2012). The relationship between self-report and lab task conceptualizations of impulsivity. *Journal of Research in Personality*, *46*(1), 121–124. <https://doi.org/10.1016/j.jrp.2011.11.005>

*Cyders, M. A., Zapolski, T. C. B., Combs, J. L., Settles, R. F., Fillmore, M. T., & Smith, G. T. (2010). Experimental effect of positive urgency on negative outcomes from risk taking and on increased alcohol consumption. *Psychology of Addictive Behaviors*, *24*(3), 367–375. <https://doi.org/10.1037/a0019494>

*Derefinko, K. J., Peters, J. R., Eisenlohr-Moul, T. A., Walsh, E. C., Adams, Z. W., & Lynam, D. R. (2014). Relations between trait impulsivity, behavioral impulsivity, physiological arousal, and risky sexual behavior among young men. *Archives of Sexual Behavior*, *43*(6), 1149–1158. <http://dx.doi.org.libproxy.berkeley.edu/10.1007/s10508-014-0327-x>

*Dhokia, M., Elander, J., Clements, K., & Gilbert, P. (2020). A randomized-controlled pilot trial of an online compassionate mind training intervention to help people with chronic pain avoid analgesic misuse. *Psychology of Addictive Behaviors*, *34*(7), 726.

*Eben, C., Chen, Z., Vermeylen, L., Billieux, J., & Verbruggen, F. (2020). A direct and conceptual replication of post-loss speeding when gambling. Royal Society open science, 7(5), 200090.

*Edge, M. D., Johnson, S. L., Ng, T., & Carver, C. S. (2013). Iowa gambling task performance in euthymic bipolar I disorder: A meta-analysis and empirical study. *Journal of Affective Disorders*, *150*(1), 115–122. <https://doi.org/10.1016/j.jad.2012.11.027>

*Flayelle, M., Verbruggen, F., Schiel, J., Vögele, C., Maurage, P., & Billieux, J. (2020). Non‐problematic and problematic binge‐watchers do not differ on prepotent response inhibition: A preregistered pilot experimental study. Human Behavior and Emerging Technologies, 2(3), 259-268.

*Gonzalez, V. M., Reynolds, B., & Skewes, M. C. (2011). Role of impulsivity in the relationship between depression and alcohol problems among emerging adult college drinkers. *Experimental and Clinical Psychopharmacology*, *19*(4), 303–313. <http://dx.doi.org.libproxy.berkeley.edu/10.1037/a0022720>

*Griffin, S. A., Lynam, D. R., & Samuel, D. B. (2018). Dimensional conceptualizations of impulsivity. *Personality Disorders: Theory, Research, and Treatment*, *9*(4), 333–345. <http://dx.doi.org.libproxy.berkeley.edu/10.1037/per0000253>

*Herman, A. M., Critchley, H. D., & Duka, T. (2019). The impact of Yohimbine-induced arousal on facets of behavioural impulsivity. *Psychopharmacology*, *236*(6), 1783–1795. <https://doi.org/10.1007/s00213-018-5160-9>

*Hlavata, P., Linhartova, P., Sumec, R., Filip, P., Svetlak, M., Balaz, M., Kasparek, T., & Bares, M. (2020). Behavioral and Neuroanatomical Account of Impulsivity in Parkinson’s Disease. *Frontiers in Neurology*, *10*, 1338. <https://doi.org/10.3389/fneur.2019.01338>

*Ho, B. C., Barry, A. B., & Koeppel, J. A. (2018). Impulsivity in unaffected adolescent biological relatives of schizophrenia patients. *Journal of Psychiatric Research*, *97*, 47–53. <https://doi.org/10.1016/j.jpsychires.2017.11.008>

*Holfelder, B., Klotzbier, T. J., Eisele, M., & Schott, N. (2020). Hot and cool executive function in elite-and amateur-adolescent athletes from open and closed skills sports. Frontiers in psychology, 11, 694.

*Jauregi, A., Kessler, K., & Hassel, S. (2018). Linking cognitive measures of response inhibition and reward sensitivity to trait impulsivity. *Frontiers in Psychology*, *9*, 2306. <http://dx.doi.org.libproxy.berkeley.edu/10.3389/fpsyg.2018.02306>

*Johnson, S. L., Tharp, J. A., Peckham, A. D., Sanchez, A. H., & Carver, C. S. (2016). Positive Urgency Is Related to Difficulty Inhibiting Prepotent Responses. *Emotion*, *16*(5), 750–759. <https://doi.org/10.1037/emo0000182>

*Kräplin, A., Dshemuchadse, M., Behrendt, S., Scherbaum, S., Goschke, T., & Bühringer, G. (2014). Dysfunctional decision-making in pathological gambling: Pattern specificity and the role of impulsivity. *Psychiatry Research*, *215*(3), 675–682. <http://dx.doi.org.libproxy.berkeley.edu/10.1016/j.psychres.2013.12.041>

*Krause-Utz, A., Cackowski, S., Daffner, S., Sobanski, E., Plichta, M. M., Bohus, M., Ende, G., & Schmahl, C. (2016). Delay discounting and response disinhibition under acute experimental stress in women with borderline personality disorder and adult attention deficit hyperactivity disorder. *Psychological Medicine*, *46*(15), 3137–3149. <http://dx.doi.org.libproxy.berkeley.edu/10.1017/S0033291716001677>

*Kvam, P. D., Romeu, R. J., Turner, B. M., Vassileva, J., & Busemeyer, J. R. (2020). Testing the factor structure underlying behavior using joint cognitive models: Impulsivity in delay discounting and Cambridge gambling tasks. *Psychological Methods*, *26*(1), 18–37. <http://dx.doi.org.libproxy.berkeley.edu/10.1037/met0000264>

*Levitt, E. E., Amlung, M. T., Gonzalez, A., Oshri, A., & MacKillop, J. (2021). Consistent evidence of indirect effects of impulsive delay discounting and negative urgency between childhood adversity and adult substance use in two samples. Psychopharmacology, 238(7), 2011-2020.

*Li, J., Weidacker, K., Mandali, A., Zhang, Y., Whiteford, S., Ren, Q., ... & Voon, V. (2021). Impulsivity and craving in subjects with opioid use disorder on methadone maintenance treatment. Drug and Alcohol Dependence, 219, 108483.

*Linhartová, P., Látalová, A., Barteček, R., ŠirÅček, J., Theiner, P., Ejova, A., Hlavatá, P., Kóša, B., JeÅábková, B., Bareš, M., & Kašpárek, T. (2019). Impulsivity in patients with borderline personality disorder: A comprehensive profile compared with healthy people and patients with ADHD. *Psychological Medicine*, *50*(11), 1829–1838. <https://doi.org/10.1017/S0033291719001892>

*Linhartová, P., Širůček, J., Ejova, A., Barteček, R., Theiner, P., & Kašpárek, T. (2019). Dimensions of Impulsivity in Healthy People, Patients with Borderline Personality Disorder, and Patients with Attention-Deficit/Hyperactivity Disorder. *Journal of Attention Disorders*, *25*(4), 584–595. <https://doi.org/10.1177/1087054718822121>

*MacKillop, J., Weafer, J., C. Gray, J., Oshri, A., Palmer, A., & de Wit, H. (2016). The latent structure of impulsivity: Impulsive choice, impulsive action, and impulsive personality traits. *Psychopharmacology*, *233*(18), 3361–3370. <https://doi.org/10.1007/s00213-016-4372-0>

*Mazza, G. L., Smyth, H. L., Bissett, P. G., Canning, J. R., Eisenberg, I. W., Enkavi, A. Z., ... & MacKinnon, D. P. (2021). Correlation database of 60 cross-disciplinary surveys and cognitive tasks assessing self-regulation. Journal of Personality Assessment, 103(2), 238-245.

*Millner, A. J., Lee, M. D., Hoyt, K., Buckholtz, J. W., Auerbach, R. P., & Nock, M. K. (2020). Are suicide attempters more impulsive than suicide ideators?. General hospital psychiatry, 63, 103-110.

*Moreno-Padilla, M., Fernández-Serrano, M. J., & Reyes del Paso, G. A. (2018). Risky decision-making after exposure to a food-choice task in excess weight adolescents: Relationships with reward-related impulsivity and hunger. *PLoS ONE*, *13*(8), e0202994. <https://doi.org/10.1371/journal.pone.0202994>

*Morris, V. L., Huffman, L. G., Naish, K. R., Holshausen, K., Oshri, A., McKinnon, M., & Amlung, M. (2020). Impulsivity as a mediating factor in the association between posttraumatic stress disorder symptoms and substance use. *Psychological Trauma: Theory, Research, Practice, and Policy*, *12*(6), 659–668. <http://dx.doi.org.libproxy.berkeley.edu/10.1037/tra0000588>

*Morrongiello, B. A., Stewart, J., Pope, K., Pogrebtsova, E., & Boulay, K.-J. (2015). Exploring relations between positive mood state and school-age children’s risk taking. *Journal of Pediatric Psychology*, *40*(4), 406–418. <http://dx.doi.org.libproxy.berkeley.edu/10.1093/jpepsy/jsu100>

*Nuyens, F., Deleuze, J., Maurage, P., Griffiths, M. D., Kuss, D. J., & Billieux, J. (2016). Impulsivity in Multiplayer Online Battle Arena gamers: Preliminary results on experimental and self-report measures. *Journal of Behavioral Addictions*, *5*(2), 351–356. <http://dx.doi.org.libproxy.berkeley.edu/10.1556/2006.5.2016.028>

*Peng-Li, D., Sørensen, T. A., Li, Y., & He, Q. (2020). Systematically lower structural brain connectivity in individuals with elevated food addiction symptoms. Appetite, 155, 104850.

*Perales, J. C., Verdejo-García, Antonio, Moya, Maribel, Lozano, Óscar, & Pérez-García, Miguel. (2009). Bright and dark sides of impulsivity: Performance of women with high and low trait impulsivity on neuropsychological tasks. *Journal of Clinical and Experimental Neuropsychology*, *31*(8), 927–944. <https://doi.org/10.1080/13803390902758793>

*Reniers, R. L. E. P., Beavan, A., Keogan, L., Furneaux, A., Mayhew, S., & Wood, S. J. (2017). Is it all in the reward? Peers influence risk‐taking behaviour in young adulthood. *British Journal of Psychology*, *108*(2), 276–295. <http://dx.doi.org.libproxy.berkeley.edu/10.1111/bjop.12195>

*Schluter, M. G., Kim, H. S., & Hodgins, D. C. (2018). Obtaining quality data using behavioral measures of impulsivity in gambling research with Amazon’s Mechanical Turk. *Journal of Behavioral Addictions*, *7*(4), 1122–1131. <https://doi.org/10.1556/2006.7.2018.117>

*Snorrason, Í., Smári, J., & Ólafsson, R. P. (2011). Motor inhibition, reflection impulsivity, and trait impulsivity in pathological skin picking. Behavior Therapy, 42(3), 521-532.

*Sofis, M. J., Budney, A. J., Stanger, C., Knapp, A. A., & Borodovsky, J. T. (2020). Greater delay discounting and cannabis coping motives are associated with more frequent cannabis use in a large sample of adult cannabis users. Drug and alcohol dependence, 207, 107820

*Squillace Louhau, M., Picon-Janeiro, J., Mazzei, N., Villar, A., & Azzollini, S. (2019). Neuropsychological Profiles of Three Subtypes of Impulsivity in the General Population: A Young Adults Study. *International Journal of Psychological Research*, *12*(1), 28–40. <https://doi.org/10.21500/20112084.3648>

*Stahl, C., Voss, A., Schmitz, F., Nuszbaum, M., Tüscher, O., Lieb, K., & Klauer, K. C. (2014). Behavioral components of impulsivity. *Journal of Experimental Psychology: General*, *143*(2), 850–886. <http://dx.doi.org.libproxy.berkeley.edu/10.1037/a0033981>

*Steward, T., Juaneda-Segui, A., Mestre-Bach, G., Martinez-Zalacain, I., Vilarrasa, N., Jimenez-Murcia, S., Fernandez-Formoso, J. A., Veciana Heras, M., Custal, N., Virgili, N., Lopez-Urdiales, R., Garcia-Ruiz-de-Gordejuela, A., Menchon, J. M., Soriano-Mas, C., & Fernandez-Aranda, F. (2019). What Difference Does it Make? Risk-Taking Behavior in Obesity after a Loss is Associated with Decreased Ventromedial Prefrontal Cortex Activity. *Journal of Clinical Medicine*, *8*(10), 1551. <https://doi.org/10.3390/jcm8101551>

*Um, M., Studebaker, A., Oglesbly, L., Sturgeon, T. & Cyders, M. (2021). The role of positive urgency in alcohol-related risk-taking. [Dissertation in preparation]. Department of Psychology, Indiana University – Purdue University Indianapolis.

*Xiao, L., Bechara, A., Grenard, L. J., Stacy, W. A., Palmer, P., Wei, Y., Jia, Y., Fu, X., & Johnson, C. A. (2009). Affective decision-making predictive of Chinese adolescent drinking behaviors. *Journal of the International Neuropsychological Society*, *15*(4), 547–557. <https://doi.org/10.1017/S1355617709090808>

*Yau, Y. H. C., Potenza, M. N., Mayes, L. C., & Crowley, M. J. (2015). Blunted feedback processing during risk-taking in adolescents with features of problematic Internet use. *Addictive Behaviors*, *45*, 156–163. <https://doi.org/10.1016/j.addbeh.2015.01.008>

*Zermatten, A., Van Der Linden, M., D’Acremont, M., Jermann, F., & Bechara, A. (2005). Impulsivity and decision making. *Journal of Nervous and Mental Disease*, *193*(10), 647–650. <https://doi.org/10.1097/01.nmd.0000180777.41295.65>

*Zhang, Y., Qiu, X., Ren, Q., Zhou, Z., Zhou, H., Du, J., Voon, V., Zhang, C., & Liu, W. (2020). Psychometric Properties of the Chinese version of UPPS-P Impulsive Behavior Scale. *Frontiers in Psychiatry*, *11*. <http://dx.doi.org.libproxy.berkeley.edu/10.3389/fpsyt.2020.00185>

*Zhu, X., Cortes, C. R., Mathur, K., Tomasi, D., & Momenan, R. (2017). Model-free functional connectivity and impulsivity correlates of alcohol dependence: A resting-state study. *Addiction Biology*, *22*(1), 206–217. <https://doi.org/10.1111/adb.12272>
